# Biogenic corrosion inhibitor on mild steel protection in concentrated HCl medium

**DOI:** 10.1038/s41598-018-20718-1

**Published:** 2018-02-08

**Authors:** Muthukumar Krishnan, Harinee Subramanian, Hans-Uwe Dahms, Vignesh Sivanandham, Palanichamy Seeni, Subramanian Gopalan, Ashok Mahalingam, Arthur James Rathinam

**Affiliations:** 1 Department of Marine Science, Bharathidasan University, Tiruchirappalli, 620 024 Tamil Nadu India; 2 Department of Physics, National Institute of Technology (NIT), Tiruchirappalli, 620 015 Tamil Nadu India; 3 Department of Biomedical Science and Environmental Biology, KMU - Kaohsiung Medical University, No.100, Shin-Chuan 1st Road, Kaohsiung, 80708 Taiwan Republic of China; 4 Research Center of Environmental Medicine, KMU - Kaohsiung Medical University, No. 100, Shin-Chuan 1st Road, Kaohsiung, 80708 Taiwan Republic of China; 5 Offshore Platform and Marine Electrochemistry Center (OPMEC), Unit of Central, Electrochemical Research Institute (CECRI), New Harbour Area, Tuticorin, 628 004 Tamil Nadu India; 6 Department of Marine Biotechnology and Resources, National Sun Yat-sen University, No. 70, Lienhai Road, Kaohsiung, 80424 Taiwan Republic of China

## Abstract

*Turbinaria ornata* (TO) extract was tested as green corrosion inhibitor on mild steel (MS) coupons in conc. HCl medium with an efficiency of 100% at 25 g l^−1^ during 5 min exposure. Antibacterial efficacy performed against 16 S rDNA identified marine biofilming bacteria (MBB) and human pathogenic bacteria (HPB). Maximum inhibition growth was 16 mm on MBB observed in *Bacillus megaterium* (MBF14 - AB894827) and 20 mm on HPB in *Escherichia coli* (B5 - NCIM 2931). Similarly, minimum of 10 mm on MBB witnessed in *Pseudomonas* sp., (MBF9 - AB894829). Toxicity studies proved 50.0% LC_50_ at 500 μg ml^−1^ in 24 hrs, whereas *Balanus amphitrite* resulted in 100% mortality within 12 hrs. Results including weight loss, potentiodynamic polarization and electrochemical impedance spectroscopy, FT-IR and GC-MS confirm 10-Octadecaonic acid as a major corrosion inhibitor from *T. ornata* and is discovered as a novel antifoulant. Anticorrosion formulation will become available soon.

## Introduction

Biofouling/biocorrosion comprises of adsorption, colonization, and undesirable accumulation of molecules and organisms on immersed substrata which may have a wide range of destructive effects on man-made structures in the aquatic environment. Biofouling is of serious concern globally in marine systems, causing considerable economic losses due to necessary maintenance and replacement operations of subsurface installations in marine technology^1^. Extra fuel is also needed in naval transportation due to increasing drag forces that also increase greenhouse gas emissions^2^.

Technologies include the development of antifouling coatings such as broad-spectrum biocides that kill or deter settling organisms. Chemical agents of organotin biocides such as tributyltin (TBT), triphenyltin (TPT), organotin compounds (OTC), or booster biocides like irgarol and diuron, heavy metals (copper oxide, zinc, arsenic and mercury oxide) were used in the past as components of effective antifoulants^3,4^. However, they are highly toxic and rapidly creating a negative impact on the aquatic environment. The Marine Environmental Protection Committee (MEPC) of the International Maritime Organization (IMO) strongly opposes the continued use of TBT, TPT or other substances which contain tin and heavy metals as biocides in antifouling paints. These were banned after 1^st^ January 2003 and the presence of such paints on surfaces of ships are completely restricted after 1^st^ January 2008^5^. For this reason, there is an urgent need to develop environmentally less harmful non-toxic antifoulant and anticorrosion paints.

Another application will be the inhibition of pathogenic microbes. Antibacterial resistances against new disease causing pathogens are on the rise in the environment. There are pathogens in the marine environment causing contagious diseases to humans and aquaculture organisms alike that could lead to high health risks and economic losses^6^. In the aquatic environment, *Bacillus subtilis* is responsible for causing food-borne gastroenteritis. *Escherichia coli, Staphylococcus aureus* and *Pseudomonas aeruginosa* cause diseases like mastitis, abortion and upper respiratory complications, while *Salmonella* spp. causes diarrhea and typhoid fever^7,8^. Antibiotic usage increased substantially recently due to an increase of infection rates and hence pathogenic bacteria became resistant to drugs, partially due to the increased and indiscriminate use of antibiotics^9^. Finding treatments against resistant pathogenic bacteria became a difficult task and the costs for drug development became more expensive. Drug application could also cause adverse effects on the host, which include hypersensitivity and depletion of beneficial microbes in the gut^10^. Decreased efficiency and resistance of pathogens to antibiotics caused the development of alternative measures. Several bioactive and pharmacologically important compounds such as alginate, carrageen and agar as phycocolloids were obtained from seaweeds and were developed to marketed drugs meanwhile. The demand to develop novel, eco-friendly antipathogenic, antifoulant and anticorrosion material is ever increasing. An attractive option in developing such materials is learning from the innovations that natural products are offering after long periods of evolutionary development.

Those natural products isolated from marine organisms that could be used as alternate agents are called synthetic antifouling coatings^11^. A number of potential antifouling compounds have been isolated and searches for antifouling compounds, often include sponges, sea plants^12^, corals^13^, ascidians^14^, sea grasses^15^, sea stars^16^, bacteria^17^, fungi^18^, micro- and macroalgae (seaweed)^3^. Among the seaweed, Phaeophyceae or brown algae play an important role in the fouling of a wide range of immersed artificial substrata. This holds particularly for shallow waters where there is sufficient light to permit the growth of algae^17^. Brown algae provide excellent bioactive/biogenic compounds exhibiting antioxidant and antifouling activities. These are belonging particularly to the group of fatty acids such as lipopeptides, amides, alkaloids, terpenoids, lactones, pyrroles and sterols^19^.

The aim of the present work is to investigate the antifouling/anticorrosion as well as antibacterial activity of three different solvent extractions (soaking and soxhlet methods in separate approaches) of ten different seaweeds. Based on antibacterial studies against marine biofilming bacteria (MBB) and human pathogenic bacteria (HPB), the effective synergistic soxhlet methanolic extract of *Turbinaria ornata* applied to mild steel (MS) on anticorrosion studies in concentrated hydrochloric acid (conc. HCl 37%) and toxicological studies against the fouling barnacle *Balanus amphitrite* and non-fouling brine-shrimp *Artemia marina* were performed. We screened ten marine seaweeds for their antifouling, anticorrosion, antibacterial activity against 16 S rDNA that belonged to MBB as well as to HPB.

## Results

### Identification of MBB

Fifteen colonies of biofilming bacteria were isolated from ship hulls where eight morphologically distinct strains were separated and cultured. The pure cultures of biofilm bacterial isolates were subjected to 16 S rDNA sequencing and the obtained multi lengths of nucleotides were subjected to a BLAST test and sequence similarity analysis by NCBI.

**Figure 1:**
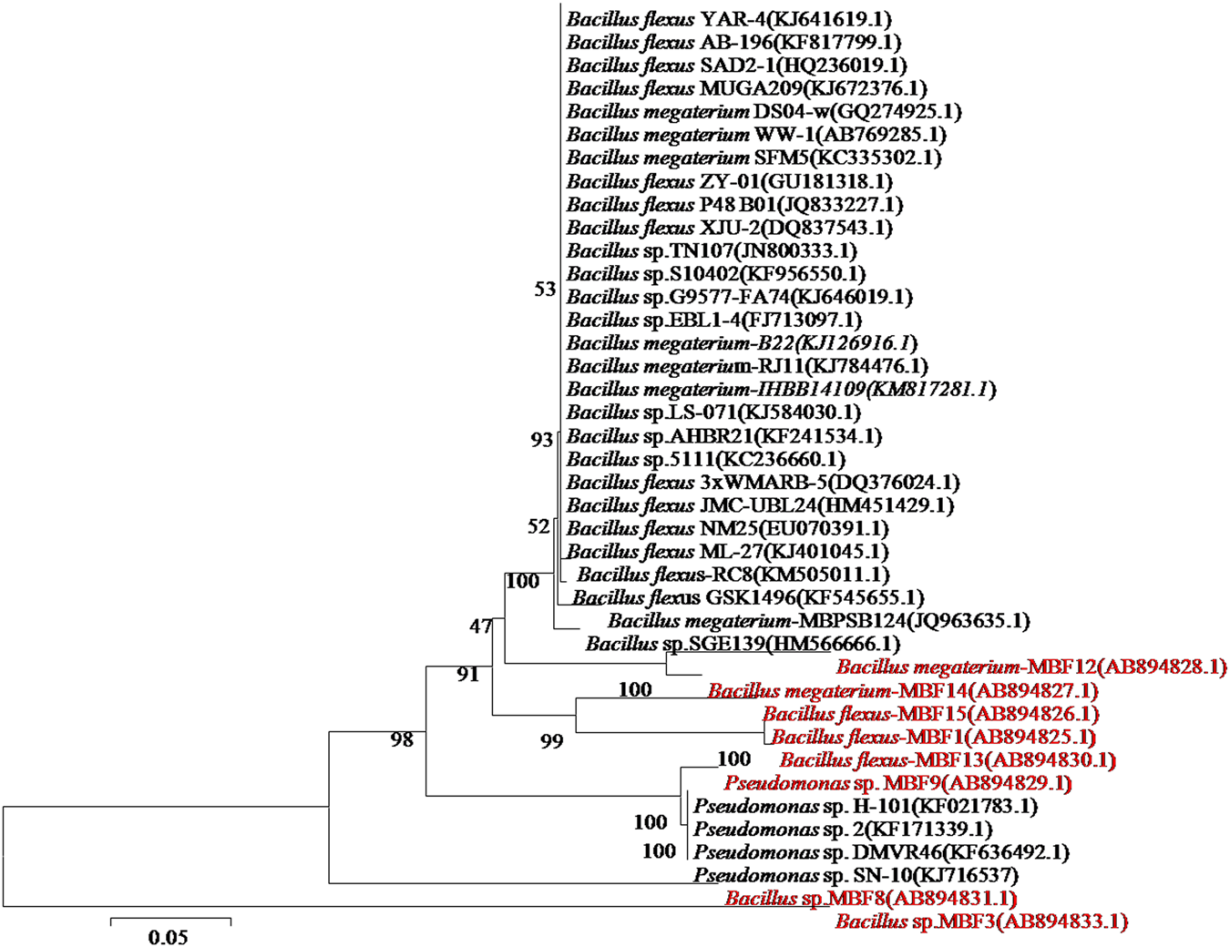
Neighbour-joining tree, based upon 16S rDNA sequences from MBB isolated from a ship hull.

Table 1 shows the results of a phylogenetic analysis based on 16 S rDNA nucleotide sequences indicating the systematic position of all isolated Gram-positive and negative MBB such as *Bacillus flexus* (MBF1 - AB894825); *Bacillus* sp., (MBF3 - AB894833); *Bacillus* sp., (MBF8 - AB894831); *Bacillus megaterium* (MBF12 - AB894828); *Bacillus flexus* (MBF13 - AB894830); *Bacillus megaterium* (MBF14 - AB894827); *Bacillus flexus* (MBF15 - AB894826); and negative as *Pseudomonas* sp., (MBF9 - AB894829) with similarity values up to 100% in Fig. 1, and Nucleotide BLAST with a non-redundant database provided in Table 2.

**Table 1:** Details of BLAST analysis, percentage of similarity and NCBI accession numbers of MBB isolated from ship hull.

S. No	Assigned code	Sequence length (bp)	Similarity (%)	BLAST results	NCBI’s accession
**Gram-positive**					
1	**MBF 1**	1429	100	*Bacillus flexus*	**AB894825**
2	**MBF 3**	1402	100	*Bacillus* sp.	**AB894833**
3	**MBF 8**	1398	100	*Bacillus* sp.	**AB894831**
4	**MBF 12**	1460	100	*Bacillus megaterium*	**AB894828**
5	**MBF 13**	1445	100	*Bacillus flexus*	**AB894830**
6	**MBF 14**	1272	100	*Bacillus megaterium*	**AB894827**
7	**MBF 15**	1295	100	*Bacillus flexus*	**AB894826**
**Gram- negative**					
8	**MBF 9**	1295	100	*Pseudomonas* sp.	**AB894829**

**Table 2:** Systematic position of the isolated MBB from ship hull.

Tag No.	Phylum	Class	Order	Family	Genus	Species
**MBF 1**	*Firmicutes*	*Bacilli*	*Bacillales*	*Bacillaceae*	*Bacillus*	*flexus*
**MBF 3**	*Firmicutes*	*Bacilli*	*Bacillales*	*Bacillaceae*	*Bacillus*	—
**MBF 8**	*Firmicutes*	*Bacilli*	*Bacillales*	*Bacillaceae*	*Bacillus*	—
**MBF 12**	*Firmicutes*	*Bacilli*	*Bacillales*	*Bacillaceae*	*Bacillus*	*megaterium*
**MBF 13**	*Firmicutes*	*Bacilli*	*Bacillales*	*Bacillaceae*	*Bacillus*	*flexus*
**MBF 14**	*Firmicutes*	*Bacilli*	*Bacillales*	*Bacillaceae*	*Bacillus*	*megaterium*
**MBF 15**	*Firmicutes*	*Bacilli*	*Bacillales*	*Bacillaceae*	*Bacillus*	*flexus*
**MBF 9**	*Proteobacteria*	*Gammaproteobacteria*	*Pseudomonadales*	*Pseudomonadacea*	*Pseudomonase*	—

(− = no species allocation).

### Antimicrobial activity of soaked seaweed extracts against MBB

Antimicrobial efficacy of soaked DEE extract of *T. ornata* (SW7) showed a maximum zone of inhibition of 13 mm against *B. flexus* (MBF13 - AB894830) and a minimum of 10 mm in *Pseudomonas* sp., (MBF9 - AB894829) in 2 mg/disc. The CHCl_3_ extract of *C. scalpelliformis* (SW4) showed, considerable antibacterial activity with a zone of inhibition of 14 mm against *Bacillus* sp., (MBF3 - AB894833) and a minimum of 10 mm in *B. megaterium* (MBF12 - AB894828). Soaking MeOH extract of *U. lactuca* (SW1) showed a maximum zone of inhibition of 13 mm against *B. flexus* (MBF1 - AB894825) and *B. megaterium* (MBF14 - AB894827) and a minimum of 10 mm in *Bacillus* sp., (MBF3 - AB894833). The effective antibacterial activate solvents against MBB were observed in the following order: CHCl_3_ > DEE = MeOH and the results were summarized in Supplementary Tables 2, 3 and 4.

### Antibacterial activity of soxhlet mediated seaweed extracts against MBB

Antibacterial activity of *C. scalpelliformis* (SW4) extracted by DEE using soxhlet showed a maximum inhibition zone (14 mm) against *B. megaterium* (MBF12 - AB894828) whereas a minimum of 10 mm was observed in *Pseudomonas* sp., (MBF9 - AB894829). Similarly, Soxhlet CHCl_3_ extract of *U. reticulata* (SW5) showed a highest zone of 15 mm against *Pseudomonas* sp., (MBF9 - AB894829) and 10 mm against *Bacillus* sp., (MBF3 - AB894833). MeOH extract of *T. ornata* (SW7) showed a larger inhibition zone of 16 mm against *B. megaterium* (MBF14 - AB894827) as well as *Pseudomonas* sp., (MBF9 - AB894829), and a smaller zone of 10 mm in *U. lactuca* (SW1) against *Bacillus* sp., (MBF3 - AB894833). Maximum antibacterial activity against MBB were high in MeOH extracts followed by CHCl_3_ and DEE extracts (Supplementary Tables 5, 6 and 7).

### Antibacterial activity of soxhlet seaweed extracts against HPB

The seaweeds, *C. antennina* (SW2) and *T. ornata* (SW7) extracted in DEE showed a major inhibition zone of 14 mm against *S. typhimurium* (B9 - NCIM 2501) and a minor of 10 mm in *S. epidermis* (B4 - NCIM 2493). At the same time, Soxhlet CHCl_3_ extracts of *S. wightii* (SW8) showed a maximum of 16 mm against *S. typhimurium* (B9 - NCIM 2501) but a minimum activity against *V. cholera* (B10 - MTCC 3906) indicated with 10 mm.

The antimicrobial efficacy of the soxhlet MeOH extract of *T. ornata* (SW7) confirmed a maximum zone of inhibition against HPB such as 10 mm in *B. subtilis* (B1 - NCIM 2920); 16 mm in *M. luteus* (B2 - NCIM 2871); 15 mm in *S. aureus* (B3 - NCIM 5021) and 10 mm in *S. epidermis* (B4 - NCIM 2493) and for Gram-negative bacteria, 20 mm was observed in *E. coli* (B5 - NCIM 2931); 18 mm in *K. pneumonia* (B6 - NCIM 2883); 14 mm in *P*. *mirabilis* (B7 - NCIM 2241); 18 mm in *P. aeruginosa* (B8 - NCIM 5029); 14 mm in *S*. *typhimurium* (B9 - NCIM 2501) and 14 mm in *V. cholera* (B10 - MTCC 3906). However, *C. antennina* (SW2) showed no activity against HPB. Antimicrobial efficacy of various seaweed resulted in the descending order: *T. ornata* (SW7) >*S. ilicifolium* (SW9) > *C. fascicularis* (SW3) > *C. scalpelliformis* (SW4) > *U. reticulata* (SW5) > *S. wightii* (SW8) > *U. lactuca* (SW1) > *G. edulis* (SW10) > *P. pavonica* (SW6) > and *C. antennina* (SW2). Similarly, maximum antibacterial activity of solvents against HPB was observed in MeOH followed by CHCl_3_ and DEE (Supplementary Tables 8, 9 and 10).

### Anti-corrosion study

Among the ten seaweeds with three different solvents (DEE, CHCl_3_ and MeOH) and two different extraction (soaking and soxhlet) methods, soxhlet MeOH extract of *T. ornata* (SW7) produced a remarkable corrosion leaching and anticorrosion activity on the MS coupons. The methanol extract from marine natural products were allowed to dry completely in a vaporized form so that the actual content of solvent was highly reduced in the medium and did not show toxicity effects. Due to its higher polarity it is one of the effective solvent used for extraction procedure and revealed ultimate antimicrobial and also non-toxic activity^20,21^.

Maximum inhibition efficiency (IE) (92.4%) was observed at low concentrations of 5.0 g l^−1^ at 10 to 15 min intervals and, thereafter, it tended to decrease. The 100% IE was achieved at maximum concentrations of 25 g l^−1^ and 30 g l^−1^ in 5 min intervals and later there were no significant decreases in the IE. This implies, the inhibitor showed a highest IE of 100% at 25 g l^−1^ in 5 min and hence this can be used for the acidization process. The results of corrosion leaching in anticorrosion experiments were presented in Table 3. The corrosion rate in blank (conc. HCl alone: M1) and the pickling solution M2 (Pickling/Clark’s solution - Stannous chloride and Antimony trioxide) recommended by ASTM is comparatively less effective than the soxhlet MeOH extracted from *T. ornata* (M3). When the concentration was increased, the anticorrosion activity also increased and hence the concentration dependence played an important role in anticorrosion processes.

**Table 3:** Inhibition efficiency (IE) of the inhibitor (soxhlet MeOH extract of *T. ornata*) for MS coupon corrosion in Conc. HCl by weight loss method.

S. No	Inhibitor concentration (g l^−1^)	Inhibition efficiency (% IE)				
		5 min	10 min	15 min	20 min	25 min
1	5 g l^−1^	87.6	92.4	92.4	90.2	87.1
2	10 g l^−1^	96.2	95.6	96.3	92.4	93
3	15 g l^−1^	98.1	98	98.8	98	97
4	20 g l^−1^	99.5	98.6	99.2	98.5	98
5	25 g l^−1^	100	99.1	99.5	99	99.4
6	30 g l^−1^	100	99.1	99.8	99.8	99.3

**Figure 2:**
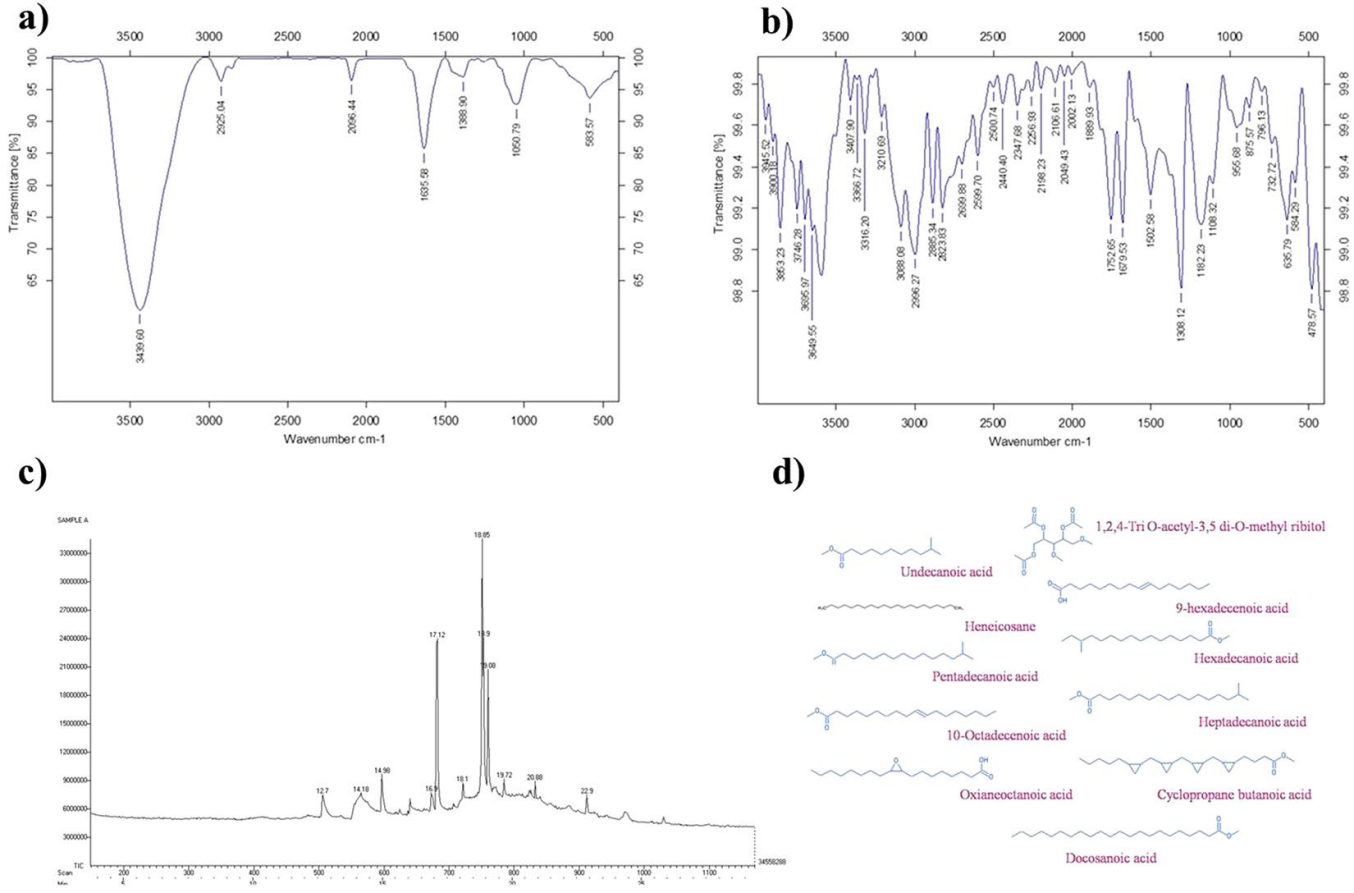
(**a**) FT-IR spectrum of the inhibitor. (**b**) FT-IR spectrum of compounds adsorbed to a MS coupon surface. (**c**) Complete GC-MS profile of soxhlet MeOH extract of *T. ornata*. (**d**) Chemical compound structures of active fraction of soxhlet MeOH extract of *T. ornata*.

Soxhlet MeOH extracts of *T. ornata* (25 g) were mixed with one liter of conc. HCl medium (25 g l^−1^) where MS coupons were immersed for 25 min and the surface of the MS coupon was studied by FT-IR spectroscopy. It shows that the inhibitor contains absorption bands at 583.57 and 1182.23 cm^−1^, corresponding to a very strong OH – stretching phenolic group and very strong asymmetric aliphatic P – O – C stretch/aromatic heteroaromatic C – H stretch. Significant peaks were shown in Figs. 2a,b. In general, the active physicochemical compounds present in the inhibitor, form a protective film on the metal surface by binding to metal ion through O, N, S or P atoms of the present functional groups. Further analyses of GC-MS on chemical characteristics of active soxhlet MeOH extracts of *T. ornata* compounds are presented in Table 4. The eleven chemical compounds from the MeOH extraction of *T. ornata* with retention times between 12.70–22.90 and the chemical structures were given in Figs. 2c,d.

**Table 4:** Analysis of secondary metabolites in *Turbinaria ornata* GC-MS details.

S. No	Peak Details	Retention Time	Area	Area %	Compound Names	Molecular Formula	Molecular Weight
1	Peak -1	12.70	11957920	5.09	Undecanoic acid	C_11_H_22_O_2_	186.29 g.mol^−1^
2	Peak -2	14.18	4269264	1.82	1,2,4-Tri O-acetyl-3,5 di-O-methyl ribitol	C_13_H_22_O_8_	306.309 g.mol^−1^
3	Peak -3	14.98	13607496	5.80	Heneicosane	C_16_H_32_O_2_	256.42 g.mol^−1^
4	Peak -4	16.90	9184204	3.91	9-hexadecenoic acid	C_16_H_30_O_2_	254.41 g.mol^−1^
5	Peak -5	17.12	54541656	23.24	Pentadecanoic acid	C_15_H_30_O_2_	242.41 g.mol^−1^
6	Peak -6	18.10	7960168	3.39	Hexadecanoic acid	C_16_H_32_O_2_	256.42 g.mol^−1^
7	Peak -7	18.85	91620384	39.04	10-Octadecenoic acid	C_19_H_36_O_2_	296.48 g.mol^−1^
8	Peak -8	19.08	29316056	12.49	Heptadecanoic acid	C_16_H_32_O_2_	256.42 g.mol^−1^
9	Peak -9	19.72	3254216	1.39	Oxianeoctanoic acid	C_18_H_34_O_3_	298.46 g.mol^−1^
10	Peak -10	20.88	5131792	2.19	Cyclopropane butanoic acid	C_3_H_6_	42.08 g.mol^−1^
11	Peak -11	22.90	3867208	1.65	Docosanoic acid	C_22_H_44_O_2_	340.58 g.mol^−1^

## Anticorrosion medium analysis

### Metal leaching analysis

After the weight loss experiment inclusive and exclusive of the inhibitor, the resultant solutions were analyzed for determination of metal iron leaching levels (leaching of Fe ions). The obtained results additionally supported our observation of weight loss. It shows a maximum metal leaching rate of 201 ppm (Fe) in 30 g l^−1^ at 5 min and 493 ppm at 25 min. This proved that with increasing inhibitor concentrations the MS coupon weights decreased in Table 5. The treated medium was processed for elemental testing by CHNS analyzer, infering the presence of carbon (40.23%), hydrogen (4.97%), nitrogen (6.42%) and sulfur (0.897%).

**Table 5:** Concentration of iron leached out due to MS coupons corrosion with and with-out the inhibitor (soxhlet extract of *T. ornata*) in Conc. HCl by weight loss solution.

S. No	Inhibitor concentration (g l^−1^)	Iron concentration in the solution (ppm)				
		5 min	10 min	15 min	20 min	25 min
1	0	38,229	43,672	49,900	57,331	89,729
2	5	5,225	5,394	5,520	8,986	11,871
3	10	1,422	3,299	2,188	2,399	3,779
4	15	981	978	848	972	1,143
5	20	895	864	599	883	978
6	25	207	490	371	1012	492
7	30	201	422	356	379	493

### Electrochemical measurements studies

**Figure 3:**
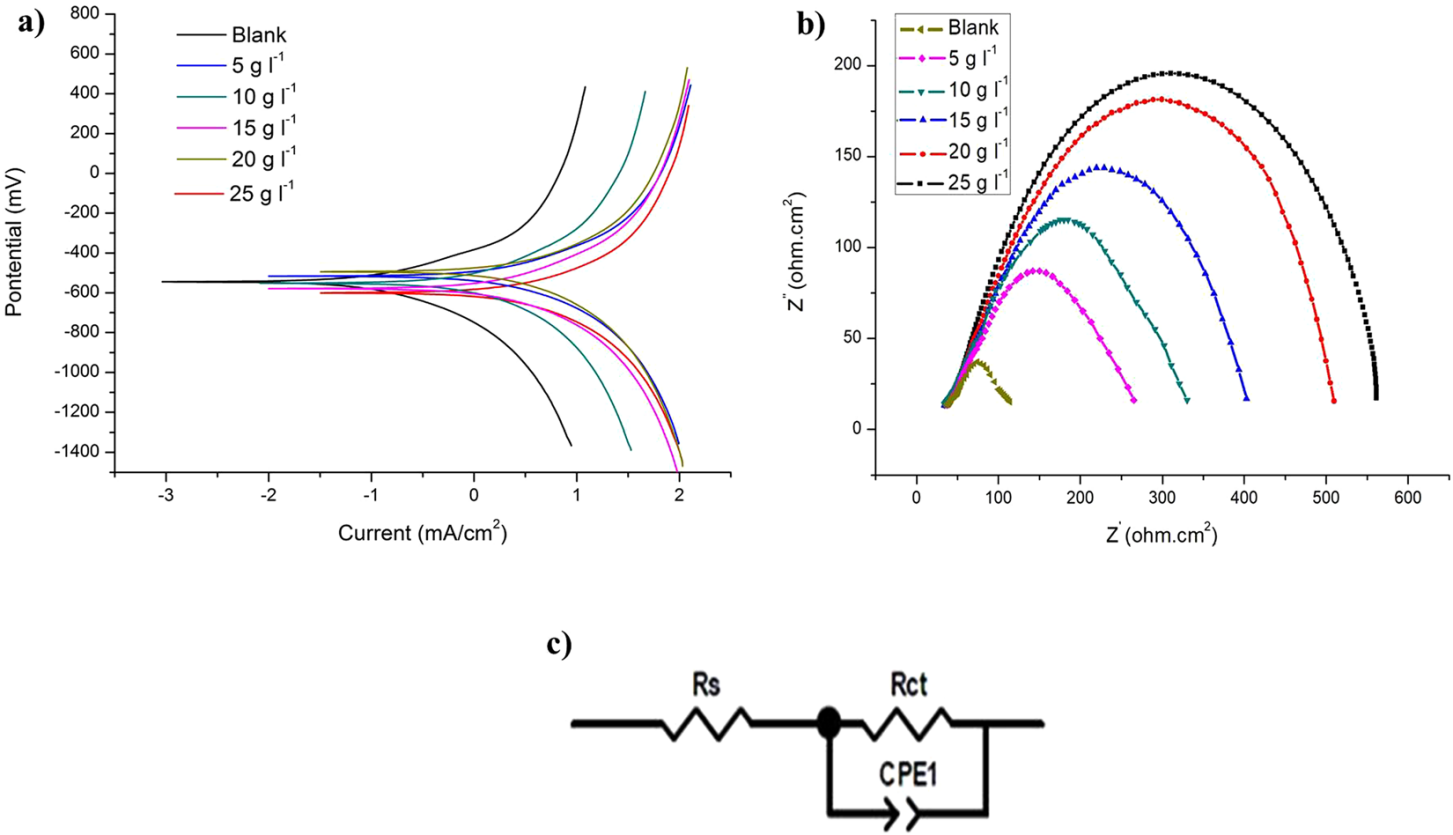
(**a**) Tafel plots of mild steel in 1 M HCl without and with containing different concentrations of soxhlet MeOH extract of *T. ornata*. (**b**) Nyquist plots of mild steel in 1 M HCl without and with different concentrations of soxhlet MeOH extract of *T. ornata*. (**c**) Equivalent circuit model used to fit the EIS data.

The potentiodynamic polarization profile of mild steel in conc. HCl solution with different concentrations of soxhlet MeOH extract of *T. ornata* are given in Fig. 3a. The numerical values of variation on the corrosion current density (I_corr_), corrosion potential (E_corr_), anodic (βa) and cathodic Tafel slope (βc) with various concentrations of soxhlet MeOH extract of *T. ornata* and corrosion-inhibition efficiency (IE) were obtained from polarization profiles and presented in Table 6. The addition of soxhlet MeOH extract of *T. ornata* caused an appreciable rise in Rp. An order of decrease in current density (I_corr_) with addition of soxhlet MeOH extract of *T. ornata* (25 g l^−1^) to the conc. HCl medium indicates a relationship between surface coverage and inhibitor concentration. The β_c_ values indicate that an addition of soxhlet MeOH extract of *T. ornata* affects both anodic and cathodic sites and hence, the soxhlet MeOH extract of *T. ornata* represents a mixed type inhibitor.

**Table 6:** Potentiodynamic polarization parameters for the corrosion of mild steel in Conc. HCl containing different concentrations soxhlet MeOH extract of *T. ornata*.

S. No	Inhibitor concentration	R_p_ (Ω cm^2^)	I_Corr_ (A cm ^−2^)	E_corr_ (V)	Tafel slopes		Inhibition efficiency (%)
					β_a_ (V dec ^−1^)	β_c_ (V dec ^−1^)	
1	0 g l^−1^	9.943	4.472 × 10^−3^	−0.499	0.1639	0.161	—
2	5 g l^−1^	21.22	9.249 × 10 ^−3^	−0.347	0.194	0.132	60.3
3	10 g ^−1^	29.19	1.267 × 10 ^−3^	−0.336	0.189	0.112	72.2
4	15 g l^−1^	38.72	9.569 × 10 ^−4^	−0.335	0.162	0.108	87.9
5	20 g l^−1^	49.99	7.921 × 10 ^−4^	−0.332	0.153	0.102	89.1
6	25 g l^−1^	61.47	6.733 × 10 ^−4^	−0.329	0.142	0.102	90.5

### Electrochemical impedance spectroscopy

In order to find the experimental results obtained from EIS measurements for corrosion of mild steel with and without inhibitor (soxhlet MeOH extract of *T. ornata*) we studied these at different concentrations. A considerable increase in total impedance was observed with addition of soxhlet MeOH extract of *T. ornata*. It can be concluded as Nyquist plots are shown in Fig. 3b that the impedance response on the mild steel was significantly altered after addition of soxhlet MeOH extract of *T. ornata* to the corrosive solution. The diameter of Nyquist plots increased with increase in inhibitor extract concentrations indicating the strengthening of the protected layer formed by inhibitor molecules. This result can be attributed to an increase in the substrate impedance with an increase in concentration of the inhibitor that is summarized in Table 7. The EIS results were analyzed using the equivalent circuit mentioned for the iron/acid thickness of the protective layer formed by inhibitor molecules which are shown in Fig. 3c.

**Table 7:** Inhibition efficiency of soxhlet MeOH extract of *T. ornata* calculated from electrochemical impedance spectroscopy (EIS) results with various concentrations.

S. No	Inhibitor concentration	R_p_ (Ω cm^2^)	R_s_ (Ω cm^2^)	R_t_ (Ω cm^2^)	Inhibition efficiency (%)
1	0 g l^−1^	4.7866	0.0942	4.6992	—
2	5 g l^−1^	17.4415	−22.971	29.35	60.3
3	10 g l^−1^	21.672	−26.142	42.106	72.2
4	15 g l^−1^	29.1042	−32.4045	71.424	89.09
5	20 g l^−1^	36.1097	−50.44	73.1091	89.24
6	25 g l^−1^	45.7801	−34.15	76.66	94.5

### Morphological study of treated and non-treated coupons

**Figure 4:**
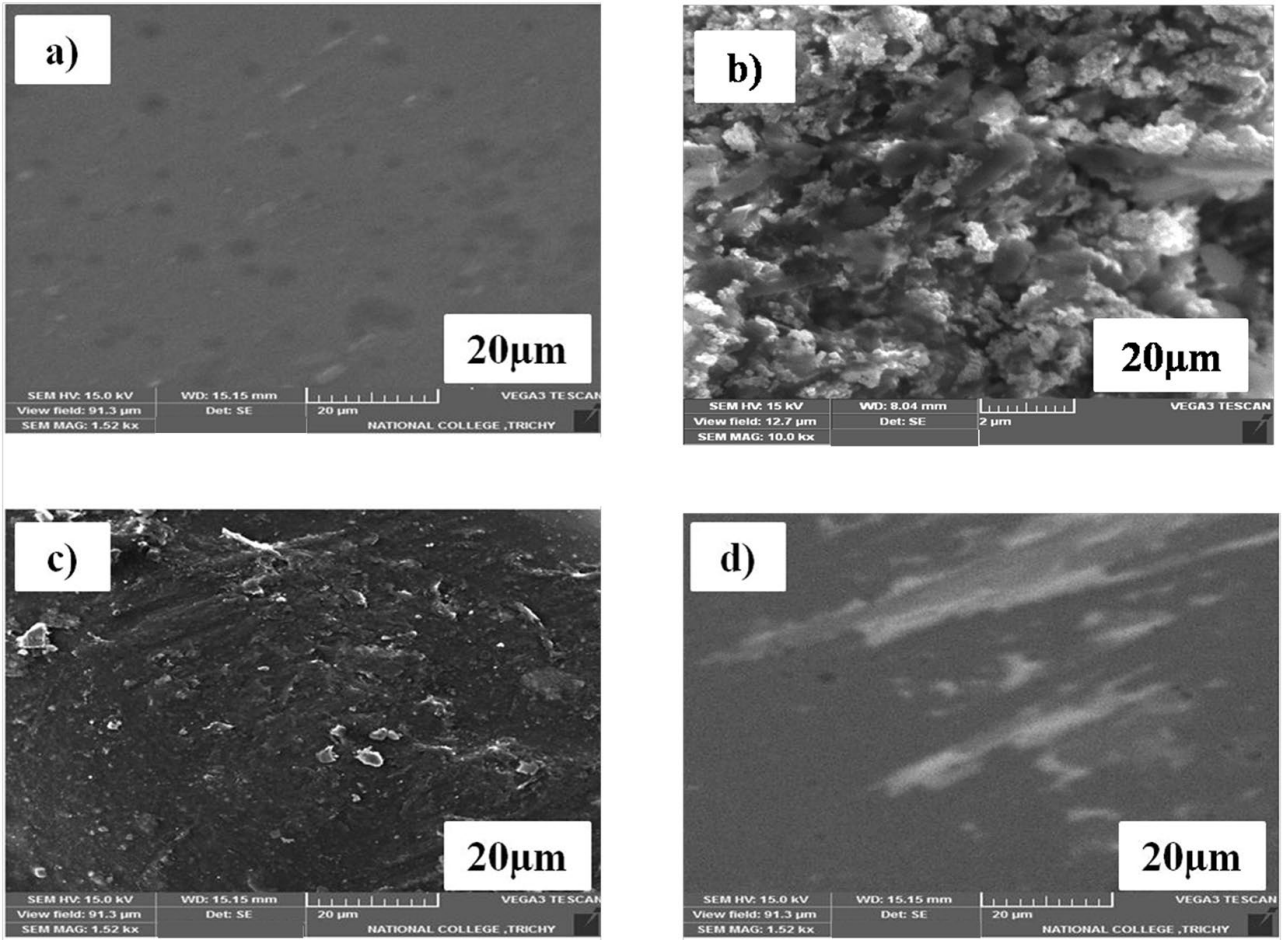
SEM photograph of MS coupons [(**a**) Polished surface, (**b**) Immersed in conc. HCl, (**c**) Immersed in standard pickling solution, (**d**) Immersed in conc. HCl with 25 g l^−1^ of the inhibitor].

**Figure 5:**
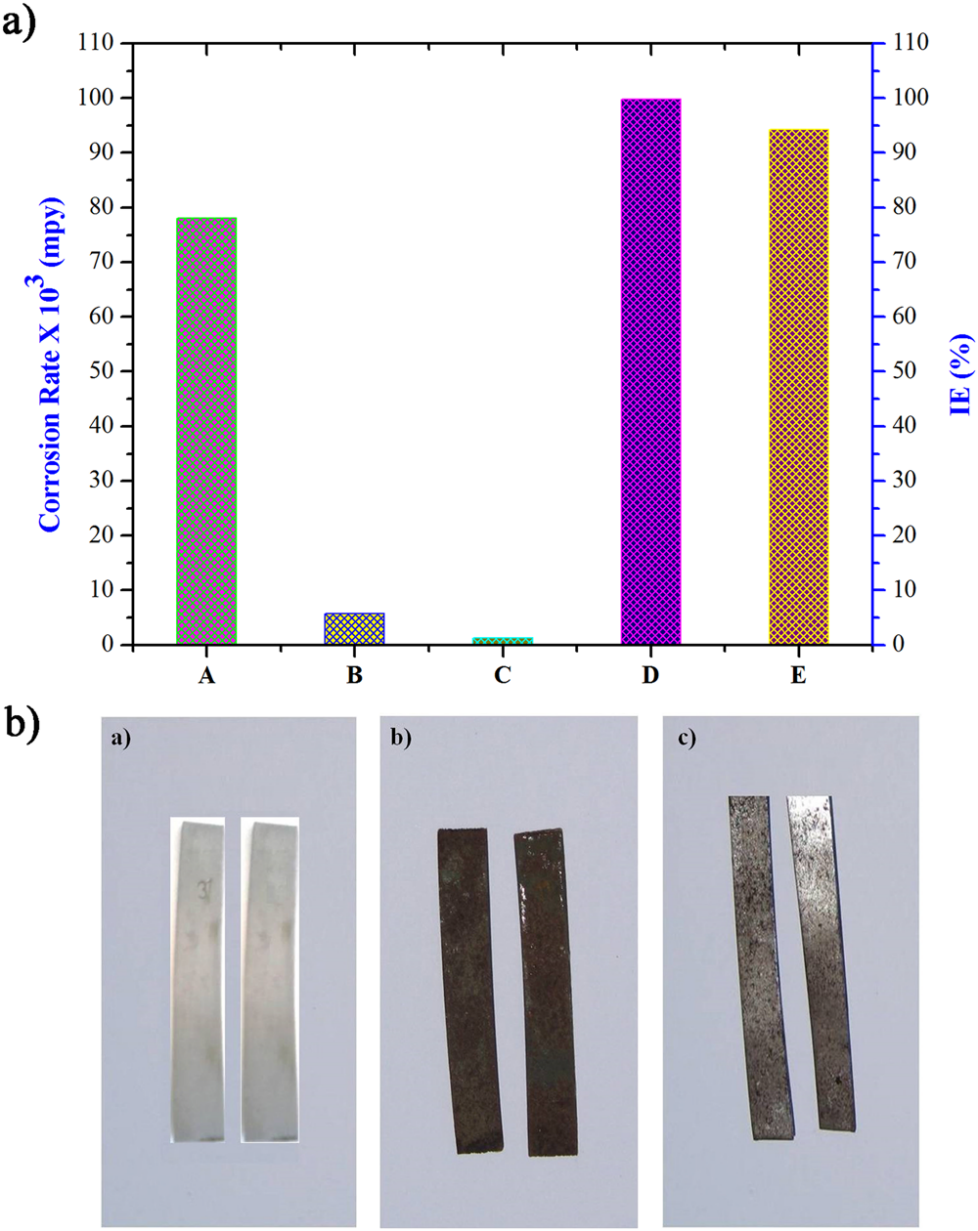
(**a**) Comparison of corrosion rate and IE of the inhibitor with standard pickling solution for MS immersed in conc. HCl for 25 min [(A) Corrosion rate for blank, (B) Corrosion rate for the inhibitor, (C) Corrosion rate for the standard pickling solution, (D) IE for the inhibitor, (E) IE for the standard pickling solution. (**b**) Comparison of corrosion rate and IE with and without the inhibitor for MS immersed in conc. HCl for 25 min [(a) Control MS coupons polished surface. (b) MS immersed in conc. HCl medium. (**c**) MS immersed in conc. HCl with 25 g l^−1^ of the inhibitor].

The different experimental approaches of treated and non-treated MS coupons were subjected to SEM analysis. The images of polished MS coupon surfaces immersed in conc. HCl solution for 25 min were shown in Figs. 4a,b. It revealed, the MS surface immersed in conc. HCl solution was severely corroded due to aggressive acid attack. The surfaces of MS coupon with pickling solution were shown in Fig. 4c as per the ASTM recommendation (1995) and the inhibitor (soxhlet MeOH extracts of *T. ornata* at 25 g l^−1^) treated MS coupon were given in Fig. 4d. It explains, adsorbed inhibitor film present on MS coupon surface, mitigated the dissolution of base metals effectively and thus experienced appreciably less corrosion than that of bare metals when compared to a pickling solution recommended by ASTM. Also, there was no significant morphological variation between the protective films formed on the MS coupon at 25 g l^−1^ of the inhibitor (Fig. 5a). When compared to polished MS coupons and inhibitor treated coupons (M3), the conc. HCl treated coupon (M1) and pickling treated coupon (M2) were highly attacked as well, indicated by their changes in morphology. Negligible base metal loss was recorded in M3 treated systems, when compared to that in M2 Clarke’s solution (Fig. 5b).

### *In-situ* detection of biofouling using phytagel

**Figure 6:**
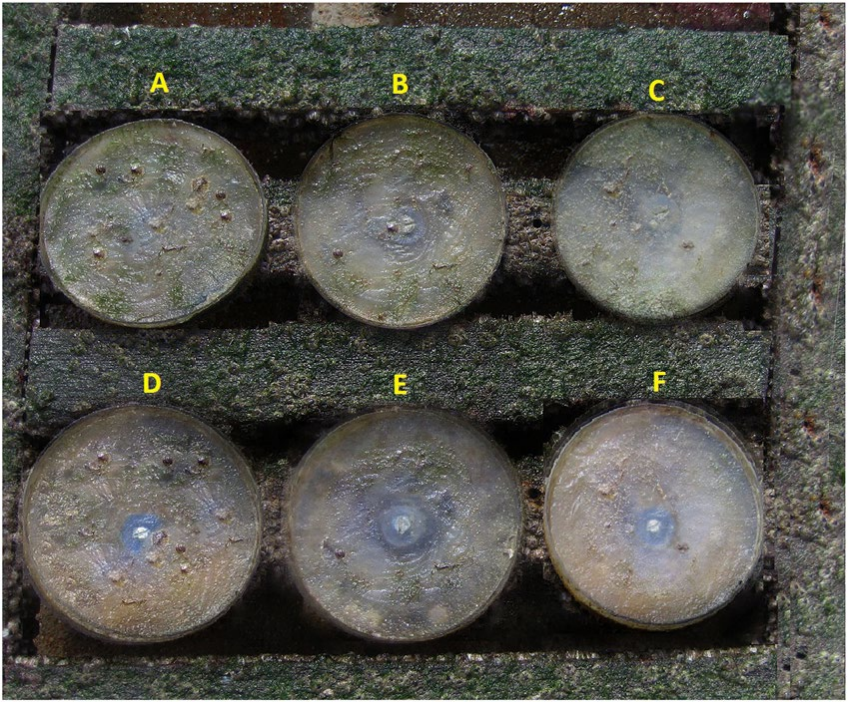
Phytagel *in-vivo* antifouling study with soxhlet MeOH extract of *T. ornata* [A) Control plates B to F) Different concentrations of *T. ornata* extract].

For an antifouling field study, a phytagel solution was mixed with five different concentrations of lyophilized soxhlet MeOH extracts of *T. ornata* and was transferred into petri dishes separately. Later, these were exposed to open seawater for a month. After incubation, the control plate was completely covered by a bacterial biofilm layer and barnacle (*Balanus amphitrite*) settlements. Interestingly, almost no micro/macro- fouling organisms were observed in the higher concentration coated petri dishes (Fig. 6). The settlement of micro- and macro-organisms was higher on the surface of the control plate than on the tested coated plates.

A mortality assay on free-swimming nauplii of *Artemia marina* was carried out with different concentrations of lyophilized soxhlet MeOH extracted *T. ornata* and this easily accumulated in the gut area without causing significant mortality in 24 hrs of exposure. Commonly, the mortality was about 10.0%. At minimum concentrations of 100 μg ml^−1^, the mortality rate was 16.6%, and as the concentration increased from 200, 300, to 400 μg ml^−1^, it reached about 23.3%, 36.6%, and 43.3%, respectively. Also, about 50.0% of the *Artemia* sp., individuals were found dead at a maximum concentration of 400 μg ml^−1^ in 48 hrs. The mortality rate was increased twice at 48 hrs of exposure and at 100 μg ml^−1^ concentration (43.3%). At concentrations of 200, 300, and 400 μg ml^−1^, it increased to 60.0%, 83.3% and 76.6%, respectively. A mortality of 96.6% was obtained at 500 μg ml^−1^ concentration (Table 8).

**Table 8:** Mortality rate (24 and 48 hours) of *Artemia* nauplii treated with various concentrations of soxhlet MeOH extract of *T. ornata* exposed to seawater an *in-vitro* study.

	Soxhlet MeOH extract of *T. ornate* (μg ml^−1^)	Initial number of *Artemia* nauplii	Number of nauplii dead after 24 hours	% of mortality after 24 hours (mean ± SD)	Number of nauplii dead after 48 hours	% of mortality after 48 hours (mean ± SD)
**Mortality rate after 24 and 48** **hours**	Control	30	3	10.00 ± 0.50	5	16.60 ± 1.52
	100	30	5	16.60 ± 0.57	13	43.30 ± 0.57
	200	30	7	23.30 ± 1.52	18	60.00 ± 1.73
	300	30	11	36.60 ± 0.57	22	73.30 ± 0.57
	400	30	13	43.30 ± 0.57	25	83.30 ± 1.15
	500	30	15	50.00 ± 1.15	29	96.60 ± 1.00

### Antifouling bioassay against *Balanus Amphitrite*

A settlement bioassay was performed to determine the antifouling properties of lyophilized soxhlet MeOH extracted *T. ornata* at different concentrations against free-swimming barnacle nauplii. 200 μg ml^−1^ concentrations of test samples at 72 hrs showed low mortality and larval settlement, whereas 400 μg ml^−1^ concentrations caused 100% larval mortality in 12 hrs which are shown in Table 9. These results indicate the tested samples were acting as potent antifouling compounds.

**Table 9:** Settlement and mortality of barnacle nauplii belonging to *Balanus amphitrite* at different concentrations of soxhlet MeOH extract of *T. ornata* and different exposure time intervals (6, 12, 24, 48 and 72 hours) exposed to seawater an *in-vitro* study.

S. No	Soxhlet MeOH extract of *T. ornata* (μg ml^−1^)	6 hours		12 hours		24 hours		48 hours		72 hours	
		Settlement (%)	Dead (%)	Settlement (%)	Dead (%)	Settlement (%)	Dead (%)	Settlement (%)	Dead (%)	Settlement (%)	Dead (%)
1	100	0	0	0	0	30	00	40	00	80	10
2	200	0	0	0	0	10	10	20	40	40	60
3	300	0	0	0	0	0	60	00	80	10	80
4	400	0	0	10	40	0	80	00	100	00	100
5	500	0	50	0	100	0	100	00	100	00	100

## Discussion

Selective utilization of seaweeds as potential sources of bioactive compounds are observed in rising numbers in recent years. Several seaweeds possess bioactive compounds and are used as curative and preventive agents on various contagious diseases such as antibiotics, antihelminthics, cough remedies, antihypertensive, antitumor, antidiarrhoeal. Limited studies are available towards reports in antibacterial, antifouling and heavy metal remediation of seaweeds belonging to Phaeophyceae (producing tannins, phenolic and other compounds)^22^. Several researchers focused on the bioactivity of algal species such as *Fucus vesiculosus*, *Sargassum natans*, *Sargassum vestitum*^23^, and *Ascophyllum nodosum*^24^. Worldwide, only a few reports are available, that finds a remedial solution on biofilm/fouling/corrosion complications in which, some of the researchers focused only on bioactive compounds from seaweeds. The need for new solutions to prevent antibacterial, antifouling and anticorrosion has considerably increased, since the prohibition of organotin additions to antifouling coatings^25–27^.

Diuron is no longer approved as a chemical antifouling and anticorrosion agent in marine paints. Respective studies show the limitation of biocides as new antifouling and anticorrosion agents. Alternatively, soft-bodied sessile marine organisms, especially macroalgae, represent a promising source of new eco-friendly antifouling and anticorrosion compounds^28^. To the best of our knowledge, the present work is first kind of report on *T. ornata* with respect to different biological and toxicological activities. Interestingly, the obtained results of both *in-vitro* and *in-vivo* studies show, MeOH solvent extracts of *T. ornata* could act as suitable eco-friendly antifoulants and anticorrosive agents. The methanol extracts of marine natural products were allowed to dry completely and the contents were redissolved in the least possible volume of methanol and used for antibacterial activity screening^29^. At the same time a control with the same concentration of methanol did not show any antibacterial activity demonstrating its non-toxic effects at the given concentration. The methanol quantity was obviously not sufficient to provide a toxic effect as well as allowed its higher polarity on extraction of important bioactive compounds from seaweeds^18,30^.

The solid-liquid extraction study showed significant variations in the concentration/weightage of crude bioactive principles from ten different seaweeds studied here. High concentration of crude bioactive compounds were obtained from MeOH extracts of *U. lactuca* (Chlorophyceae - SW1) (44.7%) through the soxhlet method whereas, lowest concentration was acquired from DEE extracts of *G. edulis* (Rhodophyceae - SW10) (10.5%) by the soaking method. The MeOH extraction of the seaweeds provided higher concentrations of bioactive principles than other solvent extractions which means, these compounds could have a higher polarity. The FT-IR and phytochemical analysis of MeOH soxhlet extracted *T. ornata* confirmed, the functional groups with high bioactivity were provided by phenolic compounds. Phenolic compounds are commonly found in plants and marine seaweeds, which have been reported to have several biological activities including antioxidant and antifouling properties.

Sixty crude solvent extracts from ten different seaweeds were tested against 18 different microorganisms (eight marine biofilming bacteria on ship hulls and ten pathogenic bacterial strains obtained from MTCC and NCIM, India). All ten seaweeds were soaked in different polarity solvents such as DEE, CHCl_3_ and MeOH. The antimicrobial efficacy of soaked (DEE, CHCl_3_ and MeOH) extracts against MBB, the CHCl_3_ extract of *C. scalpelliformis* (SW4) showed a considerable antibacterial activity of a 14 mm growth of inhibition zone against *Bacillus* sp., (MBF3 - AB894833) and lowest activity of 10 mm in *B. megaterium* (MBF12 - AB894828). In soxhlet (DEE, CHCl_3_ and MeOH) extracts against MBB, the MeOH extract of *T. ornata* (SW7) showed a higher activity against *B. megaterium* (MBF14 - AB894827) and *Pseudomonas* sp (MBF9 - AB894829) with 16 mm, whereas lower inhibition of 10 mm was observed in *U. lactuca* (SW1) against *Bacillus* sp. (MBF3 - AB894833). When compared to earlier studies^25^, a higher antimicrobial efficacy was observed in soxhlet MeOH extracts than by other solvent extracts. The present study about *B*. subtilis and *S. aureus* were correlated with earlier studies by Subba and co-workers^31^ on the brown alga *S. ilicifolium*. They reported a zone of inhibition of 10 mm against *B. subtilis* and 13 mm against *S. aureus*. The soaked MeOH extract against HPB revealed a higher activity of 20 mm in *E. coli* (B5 - NCIM 2931), whereas it was 10 mm in *B. subtilis* (B1 - NCIM 2920).

The antimicrobial efficacy of soxhlet MeOH extract of *T. ornata* (SW7) showed higher antimicrobial activity against all HPB strains than earlier reports^18^. The present study result show: 10 mm inhibition for *B. subtilis* (B1 - NCIM 2920); 16 mm in *M. luteus* (B2 - NCIM 2871); 15 mm in *S. aureus* (B3 - NCIM 5021) and 10 mm in *S. epidermis* (B4 - NCIM 2493), and on the gram-negative strains, 20 mm in *E. coli* (B5 - NCIM 2931); 18 mm in *K. pneumonia* (B6 - NCIM 2883); 14 mm in *P. mirabilis* (B7 - NCIM 2241); 18 mm in *P. aeruginosa* (B8 - NCIM 5029); 14 mm in *S. typhimurium* (B9 - NCIM 2501), and finally 14 mm in *V. cholera* (B10 - MTCC 3906). Among the obtained extracts of ten seaweeds from different methods, the soxhlet MeOH extract of *T. ornata* showed considerable antibacterial activity ranging between 10 mm to 20 mm. This seaweed could thus become a rich source of secondary metabolites and antibacterial and antifouling properties. *T. ornata* (SW7) bioactive compound are known to bind to thiol groups of bacterial DNA, RNA, affecting their proteins. Studies have demonstrated that bioactive compound interacts with sulfhydryl (SH) groups of proteins as well as the bases of DNA, leading either to respiratory inhibition or the unwinding of DNA^32^. Furthermore, *T. ornata* (SW7) extracts provided higher antimicrobial efficacy against most of the pathogens when compared to other seaweeds. Hence, *T. ornata* (SW7) could be used as an inhibitor for further anticorrosion, antifouling, toxicological and related applications.

Acid pickling and acidization are the most commonly used methods to remove/clean and desaline the corrosion related products on a metal surface. This explains why pickling is used for the cleaning and descaling of MS coupons. In order to protect the integrity of fabricated metal surfaces, corrosion inhibitors need to be applied. As part of our anticorrosion study we treated MS coupons with three different media such as natural medium as conc. HCl (blank) (M1), chemicals + blank (pickling solution - stannous chloride and antimony trioxide) (M2) and blank + test sample (soxhlet MeOH extract of *T. ornata*) (M3) were used in a weight loss study. The M3 medium was highly protecting the MS coupon from metal leaching compared to M1 and M2 medium. Adsorption isotherms are helpful in predicting the corrosion inhibition process of the inhibitor. The molecules, present in M3 render their inhibitory effect by adsorption on the MS metal surface. The adsorption of inhibitors to the metal surface is governed by several factors such as charge on the metal surface, type of corrosive media, and chemical structure of the inhibitor.

Weight loss measurements to estimate the corrosion rate and the IE of M1, M2 and M3 solution for MS coupon immersed for 25 min were carried out. Inhibitor efficiency of about 100% was achieved at 25 g l^−1^ within 5 min with no significant change (99.4%) during the study period of 25 min. It is very important to mention that the natural product chosen in the present study exhibited 100% corrosion inhibition. The American Section of the International Association for Testing Materials (ASTM-2012) suggests the weight loss method as an adequate measure of corrosion rate. This holds particularly for the inhibitory effect of an extract at higher concentration^33,34^.

A gradual decrease in corrosion rate was noticed with an increase in the concentration of a green inhibitor in a concentration dependent manner. In earlier report by Kalaiselvi *et al*.^35^ on the terrestrial plant *Artemisia pallens*, a soxhlet MeOH extract showed a maximum inhibition efficiency of about 96.4% achieved at 40 g l^−1^ in conc. HCl medium for 20 min. Here the soxhlet MeOH extracts of *T. ornata* proved minimum concentration (25 g l^−1^) with maximum inhibition efficiency (99.4%). When compared to toxic chemicals M3 [Clarke’s/pickling solution (20 g antimony trioxide and 50 g of stannous chloride were dissolved in 1000 ml of conc. HCl for 5 to 25 min at room temperature)] as per the ASTM recommendation (1995) on corrosion inhibitor, the maximum inhibition efficiency of about 92.4% was achieved in conc. HCl medium. Therefore, this green inhibitor can be an alternative against toxic chemicals in acid pickling of MS coupons.

Secondly, the MS coupons immersed in conc. HCl medium were analyzed for iron (Fe-ions) leaching from the resultant solvent which would support the notion that weight loss data of the green inhibitor were concentration dependent. Fe leaching in conc. HCl medium (M1) and pickling solution (M2) for 5 to 25 min at 29 ± 2 °C ranged between 38,229 to 89,729 ppm and 1,135 to 1645 ppm. The green inhibitor M3 at 25 g l^−1^ the observed ranges were between 201 to 493 ppm confirming that little Fe was leached and supporting the protective effect of the green inhibitor. Electrochemical results obtained from potentiodynamic polarization and EIS measurements on corrosion plots increased with an increase in inhibitor concentration. This inferred a strengthening of the protected layer formed by inhibiting molecules. In general, the acidic corrosion of mild steel was reduced by the addition of appropriate inhibitor concentration. The results attributed to increase in the substrate impedance with increase in concentration of the inhibitor. This characteristic is due to a desorption of adsorbed inhibitor molecules from mild steel surface to reduced corrosion rate^36,37^.

This was further confirmed by SEM images which revealed that the surface of MS coupon was severely corroded due to the aggressive attack by conc. HCl and mildly corroded in pickling solution treated MS coupons. However, even when a small concentration of inhibitor was added to the blank solution the MS coupon surface was more protected than the MS coupon treated by blank and pickling solution. A comparison (M3 - green inhibitor with M2 - standard pickling) of corrosion rate and IE of the inhibitor with a standard pickling solution (ASTM recommendation – 1995 & 2012) for MS coupons were performed by immersing it in conc. HCl for 20 min. In the case of a standard pickling solution for MS coupon, about 70 g of toxic chemicals such as stannous chloride and antimony trioxide are used in 1000 ml of conc. HCl.

The corrosion rate and inhibition efficiency of the inhibitor at 25 g l^−1^ was comparable with a standard pickling solution. Regarding toxicity, the LC_50_ against *Artemia* sp. value of standard pickling^38^ (stannous chloride) was 700 mg kg^−1^, whereas the LC_50_ value of soxhlet MeOH extracts of *T. ornata* resulted in 12,000 mg kg^−1^ showing that the soxhlet MeOH extract of *T. ornata* was about 20 times less toxic than the chemical inhibitor. According to Hodge and Sterner Scale^39^, the toxic rating of *T. ornata* falls in the “practically non-toxic” category. Hence, this natural/green inhibitor can be an alternative to the toxic chemicals in acid pickling of MS.

FT-IR spectrum of the inhibitor with absorption bands at 583.57 and 1182.23 cm^−1^ correspond to a very strong OH^−^ stretching phenolic group and a very strong asymmetric aliphatic P - O - C stretch/aromatic heteroaromatic C - H stretch with respective peaks. From the FT-IR and CHNS analysis, it becomes evident as the inhibitor contains all the above functional groups. Thus the inhibitive natural/green inhibitor may be attributed to synergistic effects of different functional groups adsorbed on the MS metal surface.

**Figure 7:**
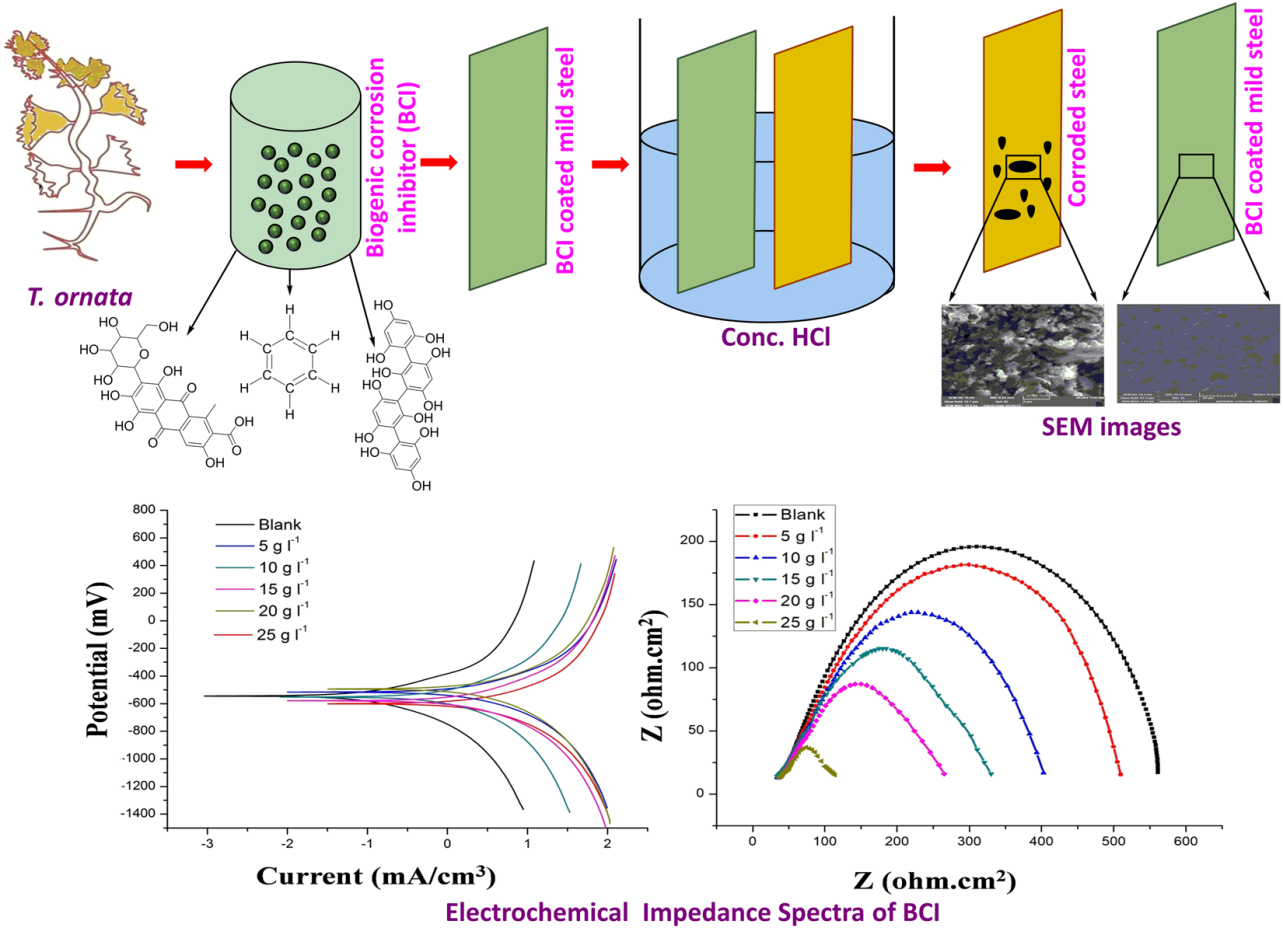
Schematic illustration on effect of biogenic corrosion inhibitor and significant anti-corrosion mechanism in Conc. HCl medium.

Studies on antifouling and anticorrosion mechanisms utilized by sessile aquatic organisms (*Artemia* and barnacle) may provide valuable information for fouling control in marine biotechnology^40^. Green extracts of *T. ornata* achieved significant mortality in 100 μg ml^−1^ concentration at 72 hrs showing low mortality and low larval settlement in barnacles, whereas, 500 μg ml^−1^ concentration caused 100% of larval mortality after 12 hrs. This indicates the potential of green extracts of *T. ornata* to act as a potent antimacrofouling compound. Both toxicity and biofilming studies confirmed that green inhibitor (MeOH extract of *T. ornata*) was controlling biofoulers without affecting other organisms such as *Artemia*. An antifouling phytagel bioassay in the field study also proved this as 2.5 g of green extract of *T. ornata* was not showing any effects on these macrofoulers (Fig. 7).

Micro/macro-organisms settled to a significantly larger extend on the surface of the control plate when compared to that of green extract of the *T. ornata* treated plate. Hornsey and Hide^41^ reported that marine algal crude extracts showed inhibitory activity against pathogenic bacteria. Differences between the results of the present investigation and results of other studies may be due to the production of different bioactive compounds and are related to season, method, organic solvents as well as differences in assay methods. Both toxicity and biofilming studies confirmed, soxhlet MeOH extract of *T. ornata* (SW7) controlled biofoulers without affecting other organisms.

Consequently, it can be concluded that the extracts of Chlorophyceae, Phaeophyceae and Rhodophyceae species showed better antibacterial activity against the MBB and HPS used. They are potential sources of bioactive compounds and should be investigated with respect to natural antibiotics. But variation in antibacterial activity may be due to the method of extraction, polarity of solvents, and the season of sampling. Thus, the higher polar solvent MeOH soxhlet extract of *T. ornata* could provide a promising candidate of MBB, HPB, corrosion inhibition of conc. HCl, and fouling (*Balanus amphitrite*) organisms as well as non-toxic (*Artemia marina*) in nature.

## Summary

Green chemistry approaches using the seaweed *T. ornata* demonstrate its potential as a natural corrosion inhibitor for MS in conc. HCl medium. The IE increased with inhibitor concentration prevents the corrosion in a dose dependent manner and was comparable with EIS data, proved inhibited solution restrain higher values than the uninhibited solution. Results from inhibitive nature of the compound in conc. HCl revealed 100% efficiency of the soxhlet MeOH extract provides a highlight in the field of natural material discovery. Mortality studies also exhibited 100% antifouling properties against *Balanus amphitrite* and toxicological studies employed *Artemia marina* demonstrate a less toxic nature of the extract to a non-fouler. Thus bioactive properties of *T. ornata* provide an alternative agent for antibacterial, antifouling, and anticorrosion coating technologies and an eco-friendly approach in future applications.

## Materials and Methods

### Collection of MBB

MBB were collected from the air-seawater interface at the bottom hull of a fishing vessel berthed at Tuticorin harbour (longitude 8° 45′ north and latitude 78° 13′ east) located at the ocean front in the new harbour area of the Gulf of Mannar, southeast coast of India. The biofilms were swab-removed using sterile cotton swabs and immediately transferred to culture tubes containing sterilized aged seawater (ASW). From the mixture (biofilm isolates + sterile aged seawater), the bacterial strains were isolated by the spread plating method (using 0.1 ml) on selective medium plates with common (10^−4^, 10^−5^, 10^−6^ and etc.) dilutions^42^. Numbers of visible colonies counted after 48 hrs of incubation in the order of magnitude above 10^4^ CFUs cm^−2^ (CFUs - Colony Forming Units). Characterization and identification of MBB was based on the results of morphologically dissimilar and distinct colonies. These were randomly selected and inoculated into rapid microbial limit test kits (Hi-media Laboratories Limited, India) for preliminary identification^43^.

### Identification of MBB based on 16S rDNA sequences

MBB were identified using the AccuPrep genomic DNA Extraction kit (Bioneer, Korea). The DNA was extracted and amplified with 16S universal primers: 27f (5′-AGA GTT TGA TCC TGG CTC AG-3′) and 1250r (5′-TAC GGT TAC CTT GTT ACG ACT T-3′). The PCR conditions were used as follows: 0.2 μM of primers, 1.5 mM of MgCl_2_, 0.2 mM of dNTPs, 0.1% of BSA (Boehringer, Mannheim, Germany), 10 ng of template DNA, and 2.5 U of *Taq* DNA polymerase were prepared in a final volume of 50 μl reaction mixture; then incubated at 95 °C for 5 min, and denatured at 94 °C for 1 min. Annealing took place at 55 °C for 1 min, extension at 72 °C for 2 min, cycles were performed for 35 times and finally 7 min extension was provided at 72 °C. The PCR products were purified using Wizard PCR Preps DNA Purification Kit (Promega) after that the size (1.5 kb) was confirmed on an agarose gel.

About 1,000 base pairs partial sequences of 16S rDNA were determined by ABI Prism Big Dye Terminator Cycle Sequencing Ready Reaction Kit (Applied Biosystems, Bagalur, India) and by an automated sequence analyzer system (model 377, Applied Biosystems, Bagalur, India). The primer set used for the sequence determination consisted of 518r (5′-GTA TTA CCG CGG CTG CTG-3′) and 338f (5′-ACT CCT ACG GGA GGC AGC-3′). The sequencing process was performed with an ABI 3130 × -Genetic Analyzer (Yaazh Xenomics, India). The obtained sequences were compared with other sequences of phylogenetically related taxa using the Basic Local Alignment Searching Tool (BLAST). Phylogenetic relationships were inferred by the neighbour-joining (NJ) method in the BLAST and sequences of the MBB were submitted to the National Center for Biotechnology Information (NCBI - Washington D.C., U.S.A.) and retrieved with an accession number^44^.

### Sequence analysis

16 S rDNA nucleotide sequences of MBB were aligned using Clustal W program version 1.5. The reference sequences were obtained from the Ribosomal Database Project (RDP) and NCBI. The base pairs with alignment uncertainty were omitted from the analysis. Phylogenetic inference was obtained by using the Bayesian approach, a model-based phylogenetic reconstruction method, as implemented in MRBAYES 3.1. This program uses the Bayes theorem, and the models of Monte Carlo Markov chains (MCMC) and the general time reversible nucleotide substitution model. The analysis was run for 1 × 10^6^ generations, and the trees were sampled after reaching chain stationary (the ‘burn-in’) and used in a majority-rule consensus tree. Full 16 S rDNA sequences of eight isolates which included both Gram-positive and negative were deposited at NCBI.

### Human pathogenic bacteria (HPB) for testing

HPB strains, which were procured such as *Bacillus subtilis* (B1 - NCIM 2920); *Micrococcus luteus* (B2 - NCIM 2871); *Staphylococcus aureus* (B3 - NCIM 5021) and *Staphylococcus epidermis* (B4 - NCIM 2493) and negative *Escherichia coli* (B5 - NCIM 2931); *Klebsiella pneumonia* (B6 - NCIM 2883); *Proteus mirabilis* (B7 - NCIM 2241); *Pseudomonas aeruginosa* (B8 - NCIM 5029); *Salmonella typhimurium* (B9 - NCIM 2501); *Vibrio cholera* (B10 - MTCC 2501). These cultures were obtained from the Council of Scientific and Industrial Research - National Chemical Industrial Microorganisms (CSIR-NCIM), Pune, India and the Council of Scientific and Industrial Research - Microbial Type of Culture Collection and Gene Bank (CSIR-MTCC), Chandigarh, India.

### Collection and preprocessing of seaweed

Fresh specimens of seaweeds belonging to Chlorophyceae, Phaeophyceae and Rhodophyceae were collected from the Mandapam coast (latitude 78° 8′ east and longitude 9° 17′ north) and Tuticorin coast (latitude 78° 13′ east and longitude 8° 45′ north) in the Gulf of Mannar region of the Bay of Bengal, southeast coast of India. The species included *Ulva lactuca* (SW1), *Chaetomorpha antennina* (SW2), *Cladophora fascicularis* (SW3), *Caulerpa scalpelliformis* (SW4), *Ulva reticulata* (SW5), *Padina pavonica* (SW6), *Turbinaria ornata* (SW7), *Sargassum wightii* (SW8), *Sargassum ilicifolium* (SW9) and *Gracilaria edulis* (SW10). The seaweed samples were hand-picked and thoroughly washed with seawater, followed by tap water and distilled water to remove adhering debris, associated epifauna and epiphytes. After cleaning, morphologically distinct thalli of the seaweeds were placed separately in sterile polyethylene bags and transported immediately to the lab and their taxonomic classifications were given in Supplementary Table 1. Fresh samples were cut into small pieces by a sterile knife and kept for a week at room temperature in the dark until further processing^45^.

### Preparation and extraction of bioactive substances from seaweeds

The different pulverized moisture-free/dried seaweeds were immersed in three different polarities like solvents such as diethyl ether (DEE), chloroform (CHCl_3_) and methanol (MeOH). Two different methods, namely soaking and soxhlet method were used for extracting the bioactive substances from the ten different seaweed species.

For the soxhlet method dried seaweed (500 g) was immersed in 1 liter of organic (DEE, CHCl_3_ and MeOH) solvents at 60 °C for 24 hrs. The extracted solute was filtered by a Millipore filter unit (0.22 μm) and it was pre-concentrated using rotary evaporation at 40 °C. Then the dried extract was dissolved in 100% of 20 ml (v/v) of the respective solvent and stored in vials for further studies^46^.

### Antimicrobial screening of MBB and HPB

*In-vitro* antimicrobial sensitivity assays were carried out using the disc diffusion method to the test samples (three different solvents and two different extraction methods of ten seaweeds) against certain MBB and HPB plated on a Muller Hinton Agar (MHA) medium. A sterile cotton swab was used to inoculate the standardized bacterial suspensions (test culture suspensions prepared in sterile 0.85% saline, matching an optical density of 0.5 McFarland standards corresponding to 10^8^ CFUs ml^−1^) on the surface of agar plates for homogeneous growth^47^. Then 15 μl of test solution (2 mg/6 mm diameter disc) were poured into a sterile disk separately, and then test sample coated discs were placed in each dish by sterilized forceps. The plates were then incubated at 37 ± 1 °C for 24–48 hrs. After incubation, the zone of inhibition was measured with a ruler/Hi Antibiotic Zone Scale-C. The assays were performed in triplicate and the average values were presented. All media, standard disks and Hi Antibiotic Zone Scale-C were purchased from Hi-Media (Mumbai, India).

### Structural analyses of active extracts

Fourier transformation infrared spectroscopy (FTIR) - Model: RX 1, Perkin Elmer range 4000 cm^−1^ to 400 cm^−1^ analysis was applied to detect bioactive molecules of seaweed extracts performed at the CSIR-CECRI, Laboratory in Karaikudi, Tamil Nadu, India. Further analyses were carried out using gas chromatography-mass spectroscopy (GC-MS) - Hewlett-Packard 5973–6890, GC-MS system (CSIR-CECRI, Laboratory in Karaikudi, Tamil Nadu, India) operating in electron ionization mode at 70 eV, equipped with a split-split less injector. The injector was set at 230 °C in a split ratio of 1:10. The column employed for analysis was a HP-5 MS fused silica capillary column (30 mm × 0.25 mm; film thickness 0.25 μm). Helium was used as a carrier gas at a flow rate of 1 ml/min. The oven temperature was 60 °C at the time of injection and was gradually raised to 250 °C at a rate of 3 °C//min and finally held at 250 °C for 10 min. The relative component concentrations were calculated from the total ion counts and identification of chemical constituents were based on the comparison of the retention time (Rt) values and mass spectra^48^.

### Corrosion inhibition study by weight loss method

MS coupons were supplied by M/s. Lawrence Metal Industries, (Chennai, India). The sizes of the coupons were 10 mm × 50 mm × 2 mm, which was used for weight loss measurements and surface analysis. The composition of MS coupons was C- 0.07%, Mn- 0.034%, P- 0.08%, and Fe- 99.8% which were measured by energy dispersive X-ray spectroscopy (EDXS) at 20 kv acceleration. The MS coupons were kept in Clarke’s/pickling solution (20 g antimony trioxide and 50 g of stannous chloride were dissolved in 1000 ml of concentrated HCl for 5 to 25 min at room temperature) as per the American Society for Testing and Materials (ASTM) recommendation (1995). Then the coupons were polished with different grades of emery papers, washed thoroughly with double distilled water and degreased with acetone^6^.

MS coupons were immersed in 100 ml of the test medium conc. HCl (37%) (M1), Clarke’s/pickling solution (M2) and conc. HCl with the addition of test samples soxhlet MeOH extract of *Turbinaria ornata* (M3) in different concentrations (5, 10, 15, 20, 25 and 30 g l^−1^). The tested coupons were removed at different time intervals (5, 10, 15, 20, and 25 min) and their weight losses were determined by using an electronic balance (Afcoset – precision ± 0.0001 g). The study was performed in triplicate and the average values were presented.

The corrosion rate in mils per year (mpy) was obtained applying the following equation:

1 $${\rm{Corrosion}}\,{\rm{rate}}\,{\rm{mpy}}=\frac{{\rm{KW}}}{{\rm{DAT}}}\times 100$$

where, K - 3.45 × 106, T - the exposure time (h), A - the surface area of the test specimen (cm^2^), W - the weight loss (g) and D - density of the test specimen (g cm^−3^).

Inhibition efficiency percentage (IE %) and the degrees of surface coverage (θ) were calculated by the following equations:

2 $${\rm{IE}}\, \% =\frac{{\rm{W}}\,-\,{{\rm{W}}}_{{\rm{1}}}}{{\rm{W}}}\times 100$$

3 $$\Theta =\frac{{\rm{W}}\,-\,{{\rm{W}}}_{{\rm{1}}}}{{\rm{W}}}$$

where, W and W1 are the weight loss of MS coupons with and without inhibitors.

### Metal leaching study

After the weight loss experiment, the solution containing medium (M3) were analyzed for Fe using an atomic absorption spectrometer (GBC-Sens AA, Australia) to measure the amount of leached trace metals (especially iron). The same test medium (M3) was also analyzed for carbon (C), hydrogen (H), and nitrogen (N) (scientific instrument - CHNS analyzer Model: Vario-EL-III) for the detection of mass fractions of carbon, hydrogen, nitrogen, heteroatom’s (X) (halogens, sulfur).

### Electrochemical measurements studies

The electrochemical measurements *viz*., potentiodynamic polarization and electrochemical impedance spectra (EIS) studies, were carried out using Autolab with PGSTAT30. The analyzer had a three-electrode cell consisting mild steel (1 cm^2^ exposure area) as the working electrode, saturated silver-silver chloride electrode as reference, and a platinum electrode as auxiliary. All the electrochemical experiments were conducted for mild steel in conc. HCl (37%) with and without conditioning in different concentrations of 5, 10, 15, 20, 25 and 30 g l^−1^ of soxhlet MeOH extract of *T. ornata* using 100 ml of test solutions in an electrical water bath fitted with temperature controller. Before each potentiodynamic polarization and electrochemical impedance study, the electrode was allowed to corrode freely and its open circuit potential (OCP) was recorded as a function of time up to 30 min after the working electrode was immersed in solution to allow steady-state potential to stabilize. And this time, the steady state OCP, corresponding to the corrosion potential of the working electrode was obtained. Each measurement was repeated three times, and only the average values were reported to verify reproducibility of the experiments. The potentiodynamic Tafel measurements were started from cathodic to the anodic direction (OCP ± 300 mV) with a scan rate of 10 mVs^−1^, and parameters such as I_corr_, E_corr_, b_a_, and b_c_ were calculated. From I_corr_, the inhibition efficiency (IE) was calculated using the following relation:

4 $${\rm{IE}}\, \% =\frac{{{\rm{I}}}_{{\rm{Corr}}}-{{\rm{I}}}_{{\rm{Corr}}(\mathrm{inh})}}{{{\rm{I}}}_{{\rm{Corr}}}}\times 100$$

where, I_Corr_ and I_Corr (inh)_ are the corrosion current densities in the absence and presence of the inhibitor, respectively.

EIS studies were performed using AC signal amplitude of 10 mV at corrosion potentials (E_Corr_) over a frequency range of 10 kHz to 10 Hz. The electrochemical parameters such as R_ct_ and C_dl_ were calculated from EIS studies using R_ct_ that was calculated by following the equation:

5 $${\rm{IE}}\, \% =\frac{{{\rm{R}}}_{{\rm{ct}}}^{0}-{{\rm{R}}}_{{\rm{ct}}}}{{{\rm{R}}}_{{\rm{ct}}}^{0}}\times 100$$

where, ${{\rm{R}}}_{{\rm{ct}}}^{0}$ and R_ct_ indicate the values of charge transfer resistances in the presence or absence of the corrosion inhibitor^49^. The corroding surface of the working electrode is expected to be inhomogeneous because of its roughness. Therefore, the capacitance is presented through a constant phase element (CPE).

### Morphological analysis of the treated coupons

The 4 different coupons used in the experiments viz. i) MS control coupon ii) MS coupons treated in conc. HCl (M1), iii) MS coupon treated in Clarke’s/pickling solution (M2) and iv) MS coupon treated with *T. ornata* extract (M3) were examined for their surface morphology using scanning electron microscopy (SEM) HITACHI, S-3000H, with a resolution of 3.5 nm. This was compared with freshly polished coupons (control, before treatment) and different MS coupon test solutions in each test division. After SEM analysis, the same MS coupons were processed for the FTIR analysis to find the particular molecules of seaweeds which adhere to the surface of test coupons.

### Mortality bioassay of *Artemia marina*

Nauplii of the brine shrimp *Artemia marina* were raised from their cysts in seawater at 29 °C and maintained for 2 days before starting the bioassay. A light source was provided to attract the free-swimming nauplii and the nauplii were transferred for the bioassay. Dried MeOH *T. ornata* 3 mg extract were dissolved in 0.6 ml of sterile triple distilled water to obtain a concentration of 5 mg ml^−1^ for the stock solution. From the stock solution, 50, 100, 150, 200 and 250 μl were placed in 5 different test tubes and the volume was filled up to 5 ml with filtered sterile seawater and the final concentration of the samples became 50, 100, 150, 200, and 250 μg ml^−1^, respectively. Approximately 30 nauplii were transferred to each test tube which had 5 ml of five different concentrations of seawater + *T. ornata* MeOH extract mixtures separately. The test tubes were incubated at 29 °C for 24–48 hrs. After incubation the test tubes were observed using magnifying hand lenses. The number of survivors, dead and immobile nauplii in each test tube were counted and noted^48^. The assays were performed in triplicate and the mean results were presented.

The percentage mortality of *Artemia marina* was calculated by the following formula:

6 $$ \% \,{\rm{Mortality}}=\frac{{\rm{Number}}\,{\rm{of}}\,{\rm{dead}}\,{Artemia}\,{nauplii}}{{\rm{Initial}}\,{\rm{number}}\,{\rm{of}}\,{\rm{live}}\,{Artemia}\,{nauplii}}\times 100$$

### Antifouling bioassay against *Balanus Amphitrite*

Adult *Balanus amphitrite* an intertidal barnacle settling on solid marine materials being approximately 1.5 cm diameter were collected in Tuticorin new harbour area, and initiated to release their nauplii in the lab using seawater at 29 °C for 2 days. A light source was used to attract the free-swimming larvae of barnacle (nauplii) and the nauplii were kept for a survival and attachment assay. An amount of 6 mg dried seaweed extract was dissolved in 1.2 ml of triple distilled water to obtain a concentration of 10 mg ml^−1^ for a stock solution. From the stock solution, 100, 200, 300, 400, and 500 μl were placed in 5 sterile glass bowls and the volumes were filled up to 10 ml with filtered seawater and the final concentration of the samples became 50, 100, 150, 200 and 250 μg ml^−1^, respectively. 5 days aged nauplii (n = 30) were transferred to each glass bowl which also contained 10 mL of five different concentrations (seawater + MeOH *T. ornata* extract mixtures). The glass bowls were incubated at 29 °C for different intervals, namely 6, 12, 24, 48 and 72 hrs. Following the different incubation times, all the glass bowls were observed for free-swimming, settled, or dead larvae. Settlement and mortality percentages were calculated to find out the efficacy of the seaweed extract as an anti-fouling agent^3,50^. All trials were carried out in triplicate and the mean values were presented.

### *In-situ* detection of biofouling using phytagel

Phytagel were procured (Sigma-Aldrich, Merck, India) into Erlenmeyer flasks containing double distilled water up to complete dissolution; then, they were placed on a hot plate at 75 °C with a magnetic stirrer. The mixture was then cooled down to 45 °C and each portion of 20 ml of phytagel solution was mixed with different concentrations (viz. 0.5, 1.0, 1.5, 2.0 and 2.5 g) of soxhlet MeOH extracts of *Turbinaria ornata*. These were poured into petri dishes (9 cm diameter) for biofouling studies. Six petri dishes added with phytagel (5 test samples were coated and one without adding any compound was used as a control) were mounted on a wooden raft using PVC washers and insulated with brass bolts and nuts. Then, the rafts were immersed in natural seawater at the Offshore Platform and Marine Electrochemistry Center (OPMEC), unit of the Central Electrochemical Research Institute (CECRI), a CSIR organization in Tuticorin (longitude 8° 45′ north and latitude 78° 13′ east), added at the ocean front in the new harbour area of the Gulf of Mannar. Phytagel coated petri dishes were removed after 30 days of immersion and biofouling organisms such as seaweed, barnacle, and oysters were identified and quantified^3,17^.

### Statement on Ethical approval

The experiments carried out in this research work are not related to live vertebrates or higher invertebrates. Hence no need to clear ethical committee clearance from our institution.

## Electronic supplementary material

Supplementary Information
