# Supplementary material for: Biogenic corrosion inhibitor on mild steel protection in concentrated HCl medium
Source: Sci Rep. 2018 Feb 8;8:2609. doi: 10.1038/s41598-018-20718-1 (PMC5805700; doi:10.1038/s41598-018-20718-1)
Supplement: Supplementary file 1 — Supplementary Information [file 41598_2018_20718_MOESM1_ESM.doc]

**SREP-17-13722**

**Biogenic corrosion inhibitor on mild steel protection in concentrated HCl medium**

Muthukumar Krishnan^1,2,4^, Harinee Subramanian^2^, Hans-Uwe Dahms^3**^, Vignesh Sivanandam^1^, Palanichamy Seeni^4^, Subramanian Gopalan^4^, Ashok Mahalingam^2^, Arthur James Rathinam^1^,*

^1^K. Muthukumar, S.Vignesh, R.A. James

Department of Marine Science, Bharathidasan University, Tiruchirappalli - 620 024,

Tamil Nadu, India.

^1,2^K. Muthukumar, S. Harinee, M. Ashok

Department of Physics, National Institute of Technology (NIT), Tiruchirappalli - 620 015,

Tamil Nadu, India.

^3^H.-U. Dahms

Department of Biomedical Science and Environmental Biology,

KMU - Kaohsiung Medical University, No.100, Shin-Chuan 1^st^ Road.

Kaohsiung 80708, Taiwan, R.O.C.;

Research Center of Environmental Medicine, KMU - Kaohsiung Medical University, No. 100, Shin-Chuan 1^st^ Road.

Kaohsiung 80708, Taiwan, R.O.C.

^1,4^K. Muthukumar, S. Palanichamy, G. Subramanian

Offshore Platform and Marine Electrochemistry Center (OPMEC), Unit of Central,

Electrochemical Research Institute (CECRI), New Harbour Area, Tuticorin - 628 004,

Tamil Nadu, India.

^1^,* Corresponding author: R. Arthur James,

Head, Department of Marine Science, Bharathidasan University,

Tiruchirappalli - 620 024, Tamil Nadu, India,

Tel: (+91)431-2407111; Fax: (+91)431-2407045,

E-mail: james@bdu.ac.in/ james.msbdu@gmail.com

^3^,** Co-corresponding author: H.-U. Dahms, Professor, Kaohsiung Medical University

Kaohsiung 80708, Taiwan.

Email: hansudahms@yahoo.com

**Table 1.** Seaweeds taxonomic classification

|  |  |  |  | **Marine Seaweeds** |  |  |
| --- | --- | --- | --- | --- | --- | --- |
| **S. No** | **Classifications** | ***Ulva lactuca***  **(SW1)** | ***Chaetomorpha antennina***  **(SW2)** | ***Cladophora Fascicularis***  **(SW3)** | **Caulerpa *scalpelliformis***  **(SW4)** | ***Ulva reticulata***  **(SW5)** |
| **1** | **Kingdom** | Plantae | Plantae | Plantae | Viridiplantae | Plantae |
| **2** | **Division** | Chlorophyta | Chlorophyta | Chlorophyta | Chlorophyta | Chlorophyta |
| **3** | **Class** | Ulvophyceae | Ulvophyceae | Ulvophyceae | Bryopsidophyceae | Ulvophyceae |
| **4** | **Order** | Ulvales | Cladophorales | Cladophorales | Bryopsidales | Ulvales |
| **5** | **Family** | Ulvaceae | Cladophoraceae | Cladophoraceae | Caulerpaceae | Ulvaceae |
| **6** | **Genus** | *Ulva* | *Chaetomorpha* | *Cladophora* | *Caulerpa* | *Ulva* |
| **7** | **Species** | *U. lactuca* | *C. antennina* | *C. fascicularis* | *C. scalpelliformis* | *U. reticulata* |
| **8** | **Binomial name** | *U. lactuca* | *C. antennina* | *C. fascicularis* | *C. scalpelliformis* | *U. reticulata* |

|  |  |  |  | **Marine Seaweeds** |  |  |
| --- | --- | --- | --- | --- | --- | --- |
| **S. No** | **Classifications** | ***Padina pavonica***  **(SW6)** | ***Turbinaria ornata***  **(SW1)** | ***Sargassum wightii***  **(SW8)** | ***Sargassum ilicifolium***  **(SW9)** | ***Gracilaria edulis***  **(SW10)** |
| **1** | **Kingdom** | Chromalveolata | Chromalveolata | Chromalveolata | Chromalveolata | Archaeplastida |
| **2** | **Division** | Heterokontophyta | Heterokontophyta | Eukaryota | Eukaryota | Eukaryota |
| **3** | **Class** | Phaeophyceae | Phaeophyceae | Phaeophyceae | Phaeophyceae | Florideophyceae |
| **4** | **Order** | Dictyotale | Fucales | Fucales | Fucales | Gracilariales |
| **5** | **Family** | Dictyotaceae | Turbinariaceae | Sargassaceae | Sargassaceae | Gracilariaceae |
| **6** | **Genus** | *Padina* | *Turbinaria* | *Sargassum* | *Sargassum* | *Gracilaria* |
| **7** | **Species** | *P. pavonica* | *T. ornata* | *S. wightii* | *S. ilicifolium* | *G. edulis* |
| **8** | **Binomial name** | *P. pavonica* | *T. ornata* | *S. wightii* | *S. ilicifolium* | *G. edulis* |

| **Inhibition zones (mm) against MBB** | | | | | | | | |
| --- | --- | --- | --- | --- | --- | --- | --- | --- |
| **Seaweed species code** | MBF1 - AB894825 | MBF3 - AB894833 | MBF8 - AB894831 | MBF12 - AB894828 | MBF13 - AB894830 | MBF14 - AB894827 | MBF15 - AB894826 | MBF9 - AB894829 |
|  |  |  |  | ***Chlorophyceae*** |  |  |  |  |
| **SW1** | 10mm | - | - | - | 10mm | - | 10mm | - |
| **SW2** | - | - | - | - | - | - | - | - |
| **SW3** | - | - | - | - | - | - | - | - |
| **SW4** | - | 11mm | 11mm | 10mm | - | 10mm | - | - |
| **SW5** | 10mm | 10mm | 10mm | - | 10mm | - | 10mm | 10mm |
|  |  |  |  | ***Phaeophyceae*** |  |  |  |  |
| **SW6** | - | - | - | - | - | - | - | - |
| **SW7** | - | - | - | - | 13mm | - | 13mm | 10mm |
| **SW8** | - | - | - | - | - | - | - | - |
| **SW9** | 10mm | - | - | - | 10mm | - | 10mm | - |
|  |  |  |  | ***Rhodophyceae*** |  |  |  |  |
| **SW10** | - | 10mm | 10mm | - | - | - | - | - |

**-** = There is no significant activity.

**SW1** – *Ulva lactuca*; **SW2** – *Chaetomorpha antennina*; **SW3** – *Cladophora fascicularis*; **SW4** – *Caulerpa scalpelliformis*; **SW5** – *Ulva reticulate*; **SW6** –

*Padina pavonica*; **SW7** – *Turbinaria ornata*; **SW8** – *Sargassum wightii*; **SW9** – *Sargassum ilicifolium* and **SW10** – *Gracilaria edulis.*

| **Inhibition zones (mm) against MBB** | | | | | | | | |
| --- | --- | --- | --- | --- | --- | --- | --- | --- |
| **Seaweed species code** | MBF1 - AB894825 | MBF3 - AB894833 | MBF8 - AB894831 | MBF12 - AB894828 | MBF13 - AB894830 | MBF14 - AB894827 | MBF15 - AB894826 | MBF9 - AB894829 |
|  |  |  |  | ***Chlorophyceae*** |  |  |  |  |
| **SW1** | 10mm | - | - | 11mm | 10mm | 11mm | 10mm | - |
| **SW2** | - | - | - | - | - | - | - | - |
| **SW3** | - | - | - | - | - | - | - | - |
| **SW4** | - | 14mm | 14mm | 10mm | - | 10mm | - | - |
| **SW5** | 11mm | 10mm | 10mm | - | 11mm | - | 11mm | 10mm |
|  |  |  |  | ***Phaeophyceae*** |  |  |  |  |
| **SW6** | - | 10mm | 10mm | - | - | --- | - | - |
| **SW7** | - | - | - | 10mm | - | 10mm | - | - |
| **SW8** | - | - | - | - | - | - | - | - |
| **SW9** | 10mm | - | - | - | 10mm | - | 10mm | - |
|  |  |  |  | ***Rhodophyceae*** |  |  |  |  |
| **SW10** | - | - | - | - | - | - | - | - |

**-** = There is no significant activity.

**SW1** – *Ulva lactuca*; **SW2** – *Chaetomorpha antennina*; **SW3** – *Cladophora fascicularis*; **SW4** – *Caulerpa scalpelliformis*; **SW5** – *Ulva reticulate*; **SW6** –

*Padina pavonica*; **SW7** – *Turbinaria ornata*; **SW8** – *Sargassum wightii*; **SW9** – *Sargassum ilicifolium* and **SW10** – *Gracilaria edulis.*

| **Inhibition zones (mm) against MBB** | | | | | | | | |
| --- | --- | --- | --- | --- | --- | --- | --- | --- |
| **Seaweed species code** | MBF1 - AB894825 | MBF3 - AB894833 | MBF8 - AB894831 | MBF12 - AB894828 | MBF13 - AB894830 | MBF14 - AB894827 | MBF15 - AB894826 | MBF9 - AB894829 |
|  |  |  |  | ***Chlorophyceae*** |  |  |  |  |
| **SW1** | 13mm | 10mm | 10mm | - | 12mm | 13mm | 12mm | - |
| **SW2** | - | - | - | - | - | - | - | - |
| **SW3** | - | - | - | - | - | - | - | - |
| **SW4** | - | 11mm | 11mm | 10mm | - | 10mm | - | - |
| **SW5** | - | 10mm | 10mm | - | - | - | - | 10mm |
|  |  |  |  | ***Phaeophyceae*** |  |  |  |  |
| **SW6** | - | 10mm | 10mm | - | - | - | - | 10mm |
| **SW7** | - | - | - | 12mm | - | 12mm | - | 12mm |
| **SW8** | - | - | - | - | - | - | - | - |
| **SW9** | 10mm | - | - | - | 10mm | - | 10mm | - |
|  |  |  |  | ***Rhodophyceae*** |  |  |  |  |
| **SW10** | 11mm | - | - | - | 11mm | - | 11mm | - |

**-** = There is no significant activity.

**SW1** – *Ulva lactuca*; **SW2** – *Chaetomorpha antennina*; **SW3** – *Cladophora fascicularis*; **SW4** – *Caulerpa scalpelliformis*; **SW5** – *Ulva reticulate*; **SW6** –

*Padina pavonica*; **SW7** – *Turbinaria ornata*; **SW8** – *Sargassum wightii*; **SW9** – *Sargassum ilicifolium* and **SW10** – *Gracilaria edulis.*

| **Inhibition zones (mm) against MBB** | | | | | | | | |
| --- | --- | --- | --- | --- | --- | --- | --- | --- |
| **Seaweed species code** | MBF1 - AB894825 | MBF3 - AB894833 | MBF8 - AB894831 | MBF12 - AB894828 | MBF13 - AB894830 | MBF14 - AB894827 | MBF15 - AB894826 | MBF9 - AB894829 |
|  |  |  |  | ***Chlorophyceae*** |  |  |  |  |
| **SW1** | - | - | - | - | - | - | - | - |
| **SW2** | - | - | 10mm | - | - | - | - | - |
| **SW3** | - | - | - | - | - | - | - | - |
| **SW4** | - | 11mm | 11mm | 14mm | - | 14mm | - | 10mm |
| **SW5** | - | 10mm | 10mm | - | - | - | - | 10mm |
|  |  |  |  | ***Phaeophyceae*** |  |  |  |  |
| **SW6** | - | - | - | - | - | - | - | - |
| **SW7** | - | - | - | - | - | - | - | - |
| **SW8** | - | - | - | - | - | - | - | - |
| **SW9** | 10mm | - | - | - | 10mm | - | 10mm | - |
|  |  |  |  | ***Rhodophyceae*** |  |  |  |  |
| **SW10** | - | 12mm | 12mm | - | - | - | - | 10mm |

**-** = There is no significant activity.

**SW1** – *Ulva lactuca*; **SW2** – *Chaetomorpha antennina*; **SW3** – *Cladophora fascicularis*; **SW4** – *Caulerpa scalpelliformis*; **SW5** – *Ulva reticulate*; **SW6** –

*Padina pavonica*; **SW7** – *Turbinaria ornata*; **SW8** – *Sargassum wightii*; **SW9** – *Sargassum ilicifolium* and **SW10** – *Gracilaria edulis.*

| **Inhibition zones (mm) against MBB** | | | | | | | | |
| --- | --- | --- | --- | --- | --- | --- | --- | --- |
| **Seaweed species code** | MBF1 - AB894825 | MBF3 - AB894833 | MBF8 - AB894831 | MBF12 - AB894828 | MBF13 - AB894830 | MBF14 - AB894827 | MBF15 - AB894826 | MBF9 - AB894829 |
|  |  |  |  | ***Chlorophyceae*** |  |  |  |  |
| **SW1** | - | - | - | - | - | - | - | - |
| **SW2** | - | 10mm | 10mm | - | - | - | - | - |
| **SW3** | - | - | - | - | - | - | - | - |
| **SW4** | - | 11mm | 11mm | 10mm | - | 10mm | - | - |
| **SW5** | 11mm | 10mm | 10mm | - | 11mm | - | 11mm | 15mm |
|  |  |  |  | ***Phaeophyceae*** |  |  |  |  |
| **SW6** | - | - | - | - | - | - | - | - |
| **SW7** | - | 10mm | 10mm | 14mm | - | 14mm | - | - |
| **SW8** | - | - | - | - | - | - | - | - |
| **SW9** | 10mm | - | - | - | 10mm | - | 10mm | - |
|  |  |  |  | ***Rhodophyceae*** |  |  |  |  |
| **SW10** | - | - | - | - | - | - | - | 10mm |

**-** = There is no significant activity.

**SW1** – *Ulva lactuca*; **SW2** – *Chaetomorpha antennina*; **SW3** – *Cladophora fascicularis*; **SW4** – *Caulerpa scalpelliformis*; **SW5** – *Ulva reticulate*; **SW6** –

*Padina pavonica*; **SW7** – *Turbinaria ornata*; **SW8** – *Sargassum wightii*; **SW9** – *Sargassum ilicifolium* and **SW10** – *Gracilaria edulis.*

| **Inhibition zones (mm) against MBB** | | | | | | | | |
| --- | --- | --- | --- | --- | --- | --- | --- | --- |
| **Seaweed species code** | MBF1 - AB894825 | MBF3 - AB894833 | MBF8 - AB894831 | MBF12 - AB894828 | MBF13 - AB894830 | MBF14 - AB894827 | MBF15 - AB894826 | MBF9 - AB894829 |
|  |  |  |  | ***Chlorophyceae*** |  |  |  |  |
| **SW1** | - | 10mm | 10mm | - | - | - | - | - |
| **SW2** | 12mm | 11mm | 11mm | - | 12mm | - | 12mm | - |
| **SW3** | - | - | - | - | - | - | - | - |
| **SW4** | - | 11mm | 11mm | 10mm | - | 10mm | - | - |
| **SW5** | - | 10mm | 10mm | - | - | - | - | 10mm |
|  |  |  |  | ***Phaeophyceae*** |  |  |  |  |
| **SW6** | - | - | - | - | - | - | - | - |
| **SW7** | 12mm | 14mm | 14mm | 14mm | 12mm | 16mm | 12mm | 10mm |
| **SW8** | - | - | - | - | - | - | - | - |
| **SW9** | 10mm | - | - | - | 10mm | - | 10mm | - |
|  |  |  |  | ***Rhodophyceae*** |  |  |  |  |
| **SW10** | 10mm | 11mm | 11mm | - | 10mm | - | 10mm | - |

**-** = There is no significant activity.

**SW1** – *Ulva lactuca*; **SW2** – *Chaetomorpha antennina*; **SW3** – *Cladophora fascicularis*; **SW4** – *Caulerpa scalpelliformis*; **SW5** – *Ulva reticulate*; **SW6** –

*Padina pavonica*; **SW7** – *Turbinaria ornata*; **SW8** – *Sargassum wightii*; **SW9** – *Sargassum ilicifolium* and **SW10** – *Gracilaria edulis.*

|  |  |  |  | **Inhibition zones (mm) against HPB** | | | |  |  |  |
| --- | --- | --- | --- | --- | --- | --- | --- | --- | --- | --- |
| **Seaweed species code** | B1 -NCIM 2920 | B2 -NCIM 2871 | B3 -NCIM 5021 | B4 - NCIM 4493 | B5 - NCIM 2931 | B6 - NCIM 2883 | B7 - NCIM 2241 | B8 - NCIM 5029 | B9 -NCIM 2501 | B10 - MTTC 3906 |
| ***Chlorophyceae*** | | | | | | | | | | |
| **SW1** | - | - | 12mm | - | - | 10mm | - | 10mm | - | - |
| **SW2** | - | - | - | 10mm | - | - | - | - | 14mm | - |
| **SW3** | 12mm | - | - | 10mm | - | - | - | - | - | - |
| **SW4** | - | - | 10mm | - | - | - | 12mm | - | - | - |
| **SW5** | - | 10mm | 10mm | - | - | 11mm | - | - | 10mm | - |
| ***Phaeophyceae*** | | | | | | | | | | |
| **SW6** | - | - | 10mm | 10mm | - | - | 10mm | - | - | 10mm |
| **SW7** | - | - | 11mm | - | 10mm | - | - | - | 14mm | - |
| **SW8** | - | - | - | 11mm | - | - | - | - | 11mm | - |
| **SW9** | - | - | 10mm | - | 11mm | - | - | 10mm | - | - |
| ***Rhodophyceae*** | | | | | | | | | | |
| **SW10** | - | 12mm | - | - | 10mm | - | - | - | - | 12mm |

|  |  |  |  | **Inhibition zones (mm) against HPB** | | | |  |  |  |
| --- | --- | --- | --- | --- | --- | --- | --- | --- | --- | --- |
| **Seaweed species code** | B1 -NCIM 2920 | B2 -NCIM 2871 | B3 -NCIM 5021 | B4 - NCIM 4493 | B5 - NCIM 2931 | B6 - NCIM 2883 | B7 - NCIM 2241 | B8 - NCIM 5029 | B9 -NCIM 2501 | B10 - MTTC 3906 |
| ***Chlorophyceae*** | | | | | | | | | | |
| **SW1** | - | - | - | - | - | - | - | - | - | - |
| **SW2** | - | - | - | - | - | - | - | - | - | - |
| **SW3** | 12mm | - | - | 10mm | - | - | - | - | - | - |
| **SW4** | - | - | 10mm | - | - | - | 12mm | - | - | - |
| **SW5** | 10mm | - | 10mm | - | - | 11mm | - | - | - | - |
| ***Phaeophyceae*** | | | | | | | | | | |
| **SW6** | - | - | 10mm | 10mm | - | - | 10mm | - | - | 10mm |
| **SW7** | 10mm | - | 12mm | - | 10mm | - | 10mm | 10mm | - | - |
| **SW8** | 12mm | 12mm | - | 14mm | - | - | - | - | 16mm | 10mm |
| **SW9** | - | - | 10mm | - | 11mm | - | - | 10mm | - | - |
| ***Rhodophyceae*** | | | | | | | | | | |
| **SW10** | - | 10mm | - | 11mm | - | - | 10mm | - | - | 10mm |

|  |  |  |  | **Inhibition zones (mm) against HPB** | | | |  |  |  |
| --- | --- | --- | --- | --- | --- | --- | --- | --- | --- | --- |
| **Seaweed species code** | B1 -NCIM 2920 | B2 -NCIM 2871 | B3 -NCIM 5021 | B4 - NCIM 4493 | B5 - NCIM 2931 | B6 - NCIM 2883 | B7 - NCIM 2241 | B8 - NCIM 5029 | B9 -NCIM 2501 | B10 - MTTC 3906 |
| ***Chlorophyceae*** | | | | | | | | | | |
| **SW1** | 10mm | - | 10mm | - | - | 10mm | - | 10mm | - | - |
| **SW2** | - | - | - | - | - | - | - | - | - | - |
| **SW3** | 12mm | 10mm | - | 10mm | - | - | - | - | 10mm | - |
| **SW4** | - | - | 10mm | - | - | - | 12mm | - | - | 12mm |
| **SW5** | 10mm | 10mm | 10mm | - | - | 11mm | - | - | - | - |
| ***Phaeophyceae*** | | | | | | | | | | |
| **SW6** | - | - | 10mm | 10mm | - | - | 10mm | - | - | 10mm |
| **SW7** | 10mm | 16mm | 15mm | 10mm | 20mm | 18mm | 14mm | 18mm | 14mm | 14mm |
| **SW8** | - | - | - | 11mm | 10mm | - | - | - | 11mm | - |
| **SW9** | 14mm | - | - | - | - | 14mm | - | - | - | - |
| ***Rhodophyceae*** | | | | | | | | | | |
| **SW10** | - | 10mm | - | - | - | - | - | - | - | 11mm |
